# Consideration of intersectoral costs and benefits in health economic evaluations of pneumococcal conjugate vaccines: a systematic review

**DOI:** 10.1017/S0266462326103912

**Published:** 2026-07-17

**Authors:** Emad Almomani, Musa Dukuray, Inge van der Putten, Aggie Paulus, Adnan Al-Lahham, Ghislaine A.P.G. van Mastrigt

**Affiliations:** 1 Department of Health Services Research, Maastricht University, Netherlands; 2 Department of Biomedical Engineering, German Jordanian University, Jordan

**Keywords:** economic evaluation, intersectoral costs and benefits, pneumococcal conjugate vaccines, pneumococcal disease, literature review

## Abstract

**Objectives:**

This systematic review explores which intersectoral costs and benefits (ICBs) are considered in the economic evaluation of pneumococcal conjugate vaccines (PCVs) across different age groups and the related impacts on the results of economic evaluations.

**Methods:**

Seven databases were searched from 2009 to 2024 for full economic evaluations (Ees) of PCVs from a societal perspective. ICBs were presented narratively and in tabular form. Studies were appraised on quality, and PRISMA guidelines were followed.

**Results:**

In all, 57 studies were included. ICBs were captured in the following sectors: patient and family, paid labor (productivity), nonpaid productivity, other sectors (education, leisure, consumption), unspecified sectors for ecological effects (herd immunity, serotype replacement, cross-protection), and equity. In cases where the comparator was no vaccination, 10 studies reported that PCVs were dominant when ICBs were included. After excluding dominant analyses, the median QALY-based incremental cost-effectiveness ratio (ICER) reduction ranged from −3.25 percent (for patient/family costs and productivity loss) to −46.02 percent (in children with ecological effects). Among DALY-based studies, the inclusion of ecological effects reduced the median ICER by −22.97 percent. In head-to-head comparisons, 16 studies reported that PCVs were dominant when ICBs were included.

**Conclusion:**

Inclusion of ICBs, except for serotype replacement, showed favorable cost-effectiveness results. This study provides a framework for identifying the intersectoral costs and benefits for the health economic evaluation of PCVs in children and adults.

## Introduction

Immunization is an important investment to reduce the health and economic burden of infectious diseases worldwide (1). Over the past decades, many countries have markedly invested in immunization programs (1;2). However, the introduction of new vaccines has remained a global concern, given budget constraints and competing investments inside and outside the healthcare sector (1). Accordingly, health economic evaluations (EEs) are used to inform decision-making about which investment to fund or reimburse in comparison with the best alternative in terms of costs and benefits (3). The impact of a vaccine (e.g., conjugate vaccine) is captured by including the benefits and externalities not only related to the treated patients but also to society. The latter, for instance, includes impacts in sectors outside the healthcare sector such as productivity gain, school attainment, and herd immunity (4). These are generally referred to as societal, multisectoral, intersectoral (5), or spillover impacts (3;6;7) or intersectoral costs and benefits (ICBs) (8). This systematic review will use the term ICBs when referring to these impacts of vaccines.

Pneumococcal diseases (PDs) are infections caused by *Streptococcus pneumoniae.* Globally, these diseases are associated with substantial health and economic burden, particularly in young children and the elderly in developing countries (9;10). The World Health Organization (WHO) reported that approximately one million children die of PDs annually (10). PDs can yield a wide range of costs in sectors outside the healthcare sector, such as education and the labor market (e.g., productivity) (11). In 2000, the 7-valent pneumococcal conjugate vaccine (PCV7) came to the market to prevent PDs in children and was first introduced in the United States. Higher-valent PCVs were licensed (e.g., 13-valent PCV) to cover additional nonvaccine serotypes and are currently available for all age groups (9;12).

Given their wide range of impacts, the prevention of PDs (e.g., conjugate vaccine implementation) can have far-reaching economic implications and may have value to decision-makers beyond the healthcare sector. Therefore, EEs of new preventative interventions, such as PCVs, require an appropriate analysis perspective (5) for measuring the broader economic impacts of vaccines (13). Some initiatives, such as the “Impact Inventory” framework, have been developed to guide researchers and address the full range of sector-specific consequences, including those outside the formal health care sector (14). Existing EE studies of PCVs, however, have traditionally focused on a narrow perspective (e.g., a healthcare perspective, only including costs and/or benefits in the healthcare sector) rather than on a broader perspective (e.g., societal, including all relevant costs and benefits with and beyond the healthcare sector). Consequently, these EEs potentially underestimate the total impact of PCVs (15).

Cross-sectoral or intersectoral economic evaluations can guide investment in health interventions, particularly in health promotion and disease prevention (16;17). However, vaccines (e.g., PCV) have distinct characteristics not shared by many other health interventions, such as herd immunity and serotype replacement (18–20). Not much is known, however, about the inclusion of ICBs of PCVs in EEs. Existing systematic reviews of PCVs have had a particular focus, such as the cost-effectiveness of PCVs in different regions (21;22), assumptions used in EEs of PCVs (23), global cost-effectiveness of PCVs (24), cost-effectiveness of PCVs in adults (25;26), the cost-effectiveness of PCV for children (27–29), and on differences in cost-effectiveness between children and the elderly population (30). However, to the best of the authors’ knowledge, evidence from systematic reviews focusing on ICBs in EEs of PCVs is lacking.

Given this research gap and the importance placed on ICBs, the objectives of this study were to: (1) explore which ICBs were considered in EEs of PCVs across different age groups and (2) assess how the inclusion of ICBs could influence the results of EEs of PCVs (e.g., in terms of their impact on the incremental cost-effectiveness ratio [ICER])

## Methods

The protocol for this systematic review was registered in PROSPERO, the International Prospective Register of Systematic Reviews database (ID: CRD42025630023). The review followed the Preferred Reporting Items for Systematic Reviews and Meta-Analyses (PRISMA) guidelines (31), Supplementary File 1, and the five-step approach for preparing a systematic review of EEs (32–34). All steps in this systematic review were undertaken by two independent reviewers (E.A and M.D). Discrepancies were resolved through discussion with a third reviewer (I.vd.P) and consensus among the review team (A.P, G.v.M, and A.A).

### Search strategy

Seven databases were searched, including MEDLINE (Ovid), Embase (Ovid), Web of Science (Core Collection), CINAHL (EBSCO), PsycINFO (EBSCO), EconLit (EBSCO), and the National Health Service Economic Evaluation Database (NHS EED) from 1 January 2009 to 31 December 2024. NHS EED was searched up to 2015 because this database no longer publishes EEs (32). PCV7 is no longer available in the global market (9). Accordingly, the year 2009 was initially selected to reflect the year of the introduction of higher valence vaccines, 10-valent (PCV10) and 13-valent (PCV13), into the global market (21).

A search string of our previous systematic review on intersectoral costs of PDs (35) was adapted and combined with relevant terms to vaccine (e.g., conjugate) and EE (e.g., cost-effectiveness) based on prior systematic reviews on EEs of PCVs (21;27–29). The full search terms can be found in Supplementary File 2. Backward and forward citations of the included studies were conducted using the online Citation Chaser tool (36).

### Inclusion and exclusion criteria

We included original studies that concerned the full EE of PCVs (e.g., cost-effectiveness analyses and cost-utility analyses) and covered higher valent PCVs containing more than 7 serotypes (e.g., PCV10, PCV13), which were compared with each other or with no vaccination in children and adults. This review considered studies that adopted or included a societal perspective in the base-case or scenario analysis, respectively. We included only human studies published in English. No restrictions were placed on the country. Studies were excluded if they compared PCV7, or 23-valent pneumococcal polysaccharide vaccine (PPSV23), or valent pneumococcal non-typeable *Haemophilus influenzae* protein D conjugate vaccine (PHiD-CV) with other available PCVs or no vaccination.

### Study selection

EndNote 21.3 was used to import search results from each database. Citations were de-duplicated using the method determined by Bramer and colleagues (37). Titles and abstracts were first screened, and then the potential full text was retrieved and reviewed to exclude irrelevant studies.

### Data extraction and analysis

A standardized data extraction sheet was utilized and adapted for this review (34). Extracted data included authors, year of publication, setting, population, intervention, comparator, analytical approach, the measured outcome, type and category of ICBs, the estimated ICER, threshold, year of valuation, currency, discount rate, the study perspective, time horizon, and the performed sensitivity analysis.

### Data analysis

ICBs were retrieved and analyzed by sector using the sector-specific classification schemes (C1–C4) developed by Drummond et al. (38): health sector (C1), other sectors (C2), patient & family (C3), and productivity (C4). This review focused on the analysis beyond the healthcare sector. Broader vaccine impacts, such as herd immunity and serotype replacement, were analyzed with inspiration from the framework by Jit et al. (2). This framework includes the following categories: A. Health-related benefits to vaccinated individuals (e.g., A1. Health gains), B. Productivity-related benefits (e.g., B1. Productivity gains related to care), and C. Community or health systems externalities (e.g., C1. Ecological effects). This review did not consider category A. Furthermore, category B will not be considered to avoid double-counting with the framework by Drummond et al. (38).

Countries were grouped into two main categories by income using the World Bank classification (39), high-income countries (HICs) and low-income, upper and lower middle-income countries (LMICs). Furthermore, the population was grouped into two main age groups: per child (≤18 years) and per adult (˃18 years). Where applicable, we explored how the inclusion of ICBs impacted the results of the ICER per sector and specific costs/benefits components. Inclusion was defined as “with versus without” analysis (40;41), for example, with ecological effects versus without, with productivity costs versus without. Ecological effects referred to herd immunity, serotype replacement, cross protection, or antimicrobial resistance.

ICERs in foreign currencies were converted to the country’s local currency using the exchange rate given in the study. If the exchange rate was not reported in the study, we used the exchange rate by the World Bank (42). ICERs were subsequently recalculated in 2024 United States dollars (USD), adjusting the values by purchasing power parity (43). When the base year of valuation was not clear, it was assumed to be the year before the study’s publication. We reported whether inclusion would decrease or increase the ICER in percentage using the median with the interquartile range (IQR). However, if less than five separate comparisons reported on specific sector or cost/benefits components, we did not report median and IQR. Furthermore, for dominant interventions, ICERS were not always reported following reporting guidelines (38). Therefore, all dominant studies were excluded from the impact analysis (41). Negative impact (%) indicates that ICERs were more favorable with inclusion of ICBs, while positive impact (%) indicates that ICERs were more negative with inclusion of ICBs. All findings are presented narratively and in tabular form.

### Quality assessment

The Consensus Health Economics Criteria List-extended (CHEC-extended) was used to appraise the quality of the economic evaluation (44). The original CHEC checklist is primarily designed for trial-based EEs (45). CHEC-extended includes an additional question applicable to model-based EEs and provides guidelines to support the value judgment. Questions were scored as “yes” (1 point), “no” (0 point), or “partial” (0.5 point). The total score per study was calculated by dividing the sum of assigned points by 20 (the total number of questions) and expressed as a percentage.

## Results

**Figure 1.**
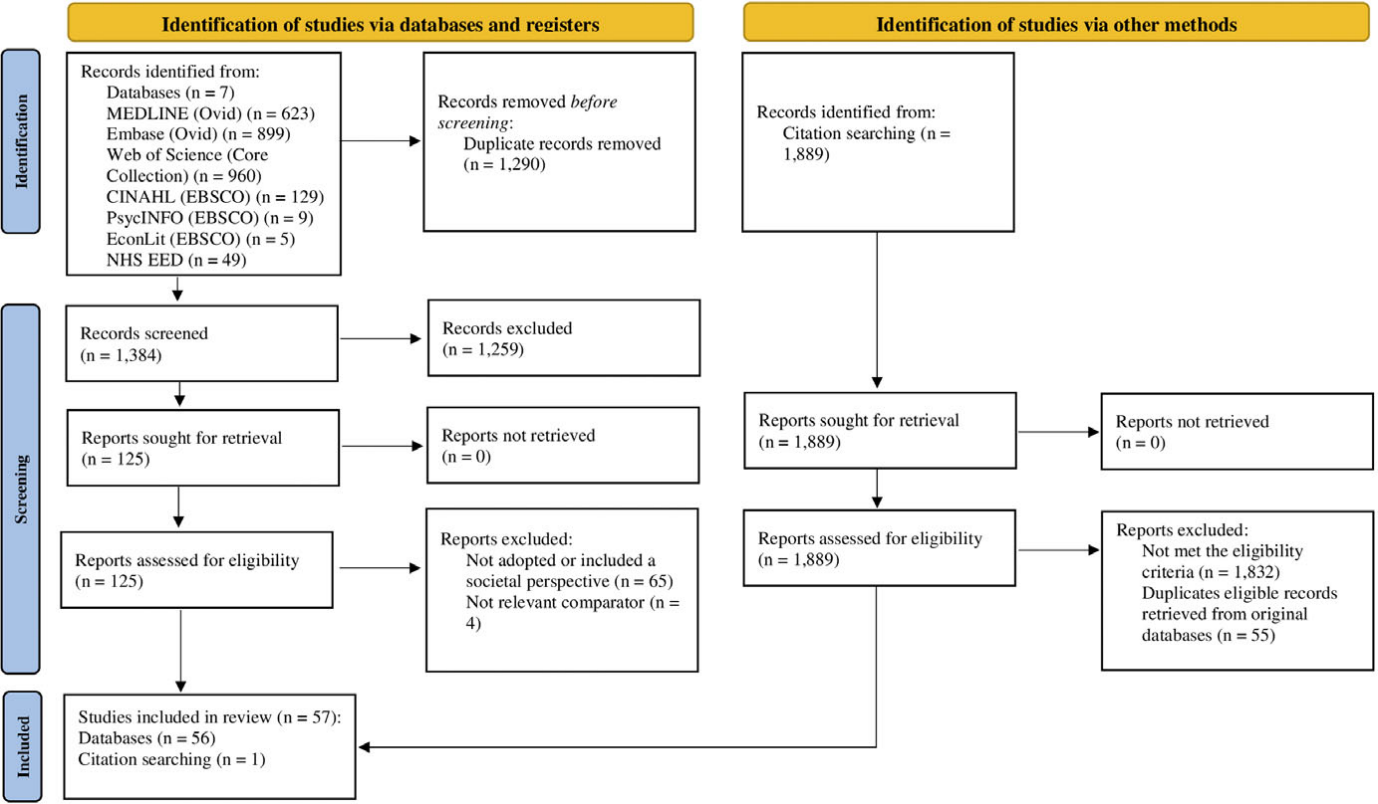
PRISMA flowchart. Figure 1. long description.

The initial search yielded a total of 1,384 studies after de-duplication. A total of 1,259 studies were excluded at the title and abstract level for the following reasons: no full EE (*n* = 547), not related to PCVs (*n* = 261), no relevant comparator (*n* = 144), not original studies (*n* = 267), non-English studies (*n* = 26), and non-human studies (*n* = 14). Then, a total of 125 studies were eligible for full-text screening; of these, fifty-six studies met the eligibility criteria. Additionally, one study was added from citation chasing. In total, fifty-seven studies (24;46–101) were included in this review (see Figure 1). Details of excluded studies after the full-text screening are provided in Supplementary File 3.

### Study characteristics

Study characteristics are summarized in Table 1. Forty-five studies reported on childhood vaccination (24;46–51;53–58;60–70;72;73;76–80;82;84–89;93–95;97–100), and twelve on adult vaccination (52;59;71;74;75;81;83;90–92;96;101). Twenty-eight studies were from high-income countries (47;52;53;55;57;59;60;64;65;71;74;75;81;84;85;87–94;96;98–101), eighteen from upper-middle-income countries (50;51;56;58;61;62;66–70;73;78–80;83;86;97), five from lower-middle-income countries (54;72;77;82;95), two from lower-income countries (46;76), and four from multiple countries with different income levels (24;48;49;63).

**Table 1.** Study characteristics of included model-based economic evaluations (*n* = 57) Table 1. long description.

Study (year)	Country (income level)	Population	Comparison	Type of approach	Type of analysis (Outcome)	Currency (year)	Time horizon	Discount rate (%)
Kim et al (2010) (46)	Gambia (LIC)	Birth cohorts	PCV9 vs NV PCV10 vs NV PCV13 vs NV	Markov (static)	CEA (DALY)	USD (2005)	5 years	3
Rozenbaum et al (2010) (47)	The Netherlands (HIC)	Newborn cohorts	PCV10 vs NV PCV13 vs NV	Decision tree (static)	CUA (QALY)	Euro (NR)	5 years, Lifetime	4 for cost, 1.5 for outcome
Nakamura et al (2011) (48)	Multiple countries (MICs)	<5 years	PCV10 vs NV PCV13 vs NV	Decision tree (static)	CEA (DALY)	USD (2005)	Lifetime	3
Tasslimi et al (2011) (49)	Multiple countries (GAVI-Eligible)	<5 years	PCV10 vs NV PCV13 vs NV	Decision tree (static)	CEA (DALY)	USD (2005)	Lifetime	3
Uruena et al (2011) (50)	Argentina (UMIC)	20 successive birth cohorts	PCV10 vs NV PCV13 vs NV PCV13 vs PCV10	TRIVAC (static)	CEA (DALY)	USD (2009)	2010–2029	3
Castaneda- Orjuela et al (2012) (51)	Colombia (UMIC)	cohort of children <1 year	PCV10 vs NV PCV13 vs PCV10	Markov	CEA (LYG)	USD (2009)	Lifetime	3
Kuhlmann et al (2012) (52)	Germany (HIC)	>50 years	PCV13 vs NV	Markov (static)	CEA (LYG)	Euro (2010)	1 year	NA
Wu et al (2012) (53)	Taiwan (HIC)	Birth cohort	PCV13 vs NV	Transmission (Dynamic)	CEA (LYG)	USD (2009)	10 years	3
Ayieko et al (2013) (54)	Kenya (LMIC)	Infants born in 2010	PCV10 vs NV PCV13 vs NV	Decision tree (static)	CEA (DALY, Death, Case)	USD (2010)	NR	3
Hoshi et al (2013) (55)	Japan (HIC)	Birth cohorts	PCV13 vs NV	Markov (static)	CUA (QALY), CEA (YLOS)	JPY (NR)	5 years	3
Kulpeng et al (2013) (56)	Thailand (UMIC)	2–15 months	PCV10 vs NV PCV13 vs NV	Markov (static)	CUA (QALY)	THB (2010)	Lifetime	3
Vemer & Postma (2014) (57)	The Netherlands (HIC)	Birth cohort	PCV13 vs PCV10	Decision tree (static)	CUA (QALY)	Euro (2012)	5 years	4 for cost, 1.5 for outcome
Kieninger et al (2015) (58)	Paraguay (UMIC)	<5 years	PCV10 vs NV PCV13 vs NV PCV13 vs PCV10	TRIVAC (static)	CEA (DALY)	USD (2009)	10 years	3
Mangen et al (2015) (59)	The Netherlands (HIC)	65–74 years	PCV13 vs NV	Markov	CEA (LYG), CUA (QALY)	Euro (2012)	Lifetime	4 for cost, 1.5 for outcome
Vucina et al (2015) (60)	Croatia (HIC)	20 successive birth cohorts	PCV10 vs NV PCV13 vs NV PCV13 vs PCV10	TRIVAC (static)	CEA (DALY)	USD (2014)	2014–2033	3
Maurer et al (2016) (61)	China (UMIC)	cohort of 16 million infants	PCV10 vs NV PCV13 vs NV	Markov	CUA (QALY)	USD (2014)	Lifetime	3
Mo et al (2016) (62)	China (UMIC)	100,000 newborns	PCV13 vs NV	Markov (static)	CUA (QALY)	USD (2015)	100 years	3
Wu et al (2016) (63)	Malaysia (UMIC), Hong Kong (HIC)	10 successive birth cohorts	PCV10 vs NV PCV13 vs NV PCV13 vs PCV10	Markov (static)	CUA (QALY)	USD (2014)	10 years	3
Gouveia et al (2017) (64)	Portugal (HIC)	100,000 newborns	PCV13 vs NV	Markov (static)	CEA (LYG)	Euro (2015)	Lifetime	5
Kuhlmann & von der Schulenburg (2017) (65)	Germany (HIC)	<2 years	PCV13 vs PCV10	Markov (static)	CEA (LYG), CUA (QALY)	Euro (2013)	50 years	3
Sundaram et al (2017) (66)	Mongolia (UMIC)	30 birth cohorts	PCV13 vs NV	Decision tree (static)	CEA (DALY)	USD (2014)	30 years	3
Lara et al (2018) (67)	Colombia (UMIC)	<5 years	PCV10 vs NV	Markov	CEA (DALY)	I$ (2008)	2008–2012	3
Zhou et al (2018) (68)	China (UMIC)	birth cohort	PCV13 vs NV	Markov (static)	CUA (QALY)	USD (2016)	Lifetime	5
Chen et al (2019) (24)	Multiple countries (Global)	30 birth cohorts	PCV13 vs NV	Ecological and Decision tree (dynamic)	CEA (DALY)	I$ (2015)	30 years	3
Dilokthornsakul et al (2019) (69)	Thailand (UMIC)	birth cohort	PCV10 vs NV PCV13 vs NV	Markov (static)	CUA (QALY)	THB (2017)	Lifetime	3
Ezoji et al (2019) (70)	Iran (UMIC)	<5 years	PCV13 vs NV	TRIVAC (static)	CEA (DALY)	USD (2014)	10 years	3
Gouveia et al (2019) (71)	Portugal (HIC)	≥18 years	PCV13 vs NV	Markov (static)	CEA (LYG), CUA (QALY)	Euro (2013)	Lifetime	5
Idris et al (2020) (72)	Nigeria (LMIC)	<5 years	PCV13 vs NV	UNIVAC (static)	CEA (DALY)	USD (2017)	20 years	3
Shafie et al (2020) (73)	Malaysia (UMIC)	<2 years	PCV13 vs NV PCV13 vs PCV10	Decision analytic (static)	CUA (QALY)	USD (2018)	5 years	3
de Vries et al (2021) (74)	The Netherlands (HIC)	≥18 years	PCV13 vs NV	Model-based (static)	CUA (QALY)	Euro (2017)	15 years	4 for cost
Igarashi et al (2021) (75)	Japan (HIC)	≥60 years	PCV13 vs NV	Markov microsimulation (dynamic)	CEA (LYG), CUA (QALY)	JPY (2021)	Lifetime	2
Pecenka et al (2021) (76)	Gambia (LIC)	20 birth cohorts	PCV13 vs NV	UNIVAC (static)	CEA (DALY)	USD (2017)	20 years	3
Perdrizet et al (2021) (77)	Philippines (LMIC)	<2 years	PCV13 vs PCV10	Decision analytic (static)	CUA (QALY)	PHP (2020)	10 years	7
Suwantika et al (2021) (78)	Indonesia (UMIC)	<5 years	PCV10 vs NV PCV13 vs NV	Decision tree (static)	CUA (QALY)	USD (2019)	5 years	3
Wang et al (2021) (79)	China (UMIC)	<5 years	PCV13 vs NV	Markov (static)	CUA (QALY)	USD (2019)	Lifetime	5
Chaiyakunapruk et al (2022) (80)	Thailand (UMIC)	<6 months	PCV10 vs NV PCV12 vs NV PCV13 vs NV	Markov (static)	CUA (QALY)	THB (NR)	Lifetime	3
Deb et al (2022) (81)	Switzerland (HIC)	≥18 years	PCV15 vs NV	Markov (static)	CUA (QALY)	CHF (2020)	Lifetime	3
Sevilla et al (2022) (82)	Egypt (LMIC)	100 successive birth cohorts	PCV10 vs NV PCV13 vs NV PCV13 vs PCV10	Markov, (static)	CUA (QALY), CBA (ROR)	USD (2016)	100 years	3
Guo et al (2023) (83)	China (UMIC)	≥65 years	PCV13 vs NV	Markov (static)	CUA (QALY)	USD (2019)	Lifetime	3
Huang et al (2023) (84)	United States (HIC)	<2 years	PCV15 vs PCV13	Markov (static)	CEA (LYs), CUA (QALY)	USD (2021)	100 years	3
Lytle et al (2023) (85)	Canada (HIC)	<2 years	PCV20 vs PCV13 PCV20 vs PCV15	Markov (static)	CUA (QALY)	CAD (2022)	10 years	1.5
Ordonez & Ordonez (2023) (86)	Colombia (UMIC)	<5 years	PCV13 vs PCV10	Decision tree (dynamic)	CEA (LYG)	USD (2022)	80 years	5
Prasad et al (2023) (87)	United States (HIC)	birth cohort	PCV15 vs PCV13	NR	CEA (LYG), CUA (QALY)	USD (2021)	15 years	3
Tajima et al (2023) (88)	Japan (HIC)	birth cohort	PCV15 vs PCV13	Markov (static)	CEA (LYG), CUA (QALY)	JPY (2022)	10 years	2
Wilson et al (2023) (89)	United Kingdom (HIC)	UK children	PCV15 vs PCV13 PCV20 vs PCV13 PCV20 vs PCV15	Dynamic transmission	CUA (QALY)	GBP (2021)	5 years	3.5
Altawalbeh et al (2024) (90)	United States (HIC)	≥50 years	PCV21 vs NV PCV21 vs PCV20	Markov	CUA (QALY)	USD (2019)	Lifetime	3
Altawalbeh et al (2024) (91)	United States (HIC)	≥50 years	PCV20 vs NV	Markov	CUA (QALY)	USD (NR)	Lifetime	3
de Boer et al (2024) (92)	The Netherlands (HIC)	≥65 years	PCV15 vs NV PCV20 vs NV PCV21 vs NV	Decision tree (static)	CUA (QALY)	Euro (2021)	15 years	4 for cost, 1.5 for outcome
Fridh et al (2024) (93)	Sweden (HIC)	<2 years	PCV20 vs PCV15	Markov (static)	CUA (QALY)	SEK (2023)	10 years	3
Huang et al (2024) (94)	Germany (HIC)	birth cohort	PCV20 vs PCV15	Markov (static)	CUA (QALY)	Euro (2023)	Lifetime	3
Ibrahim et al (2024) (95)	Ghana (LMIC)	20 successive Birth cohorts	PCV13 vs NV	UNIVAC (static)	CEA (DALY)	USD (2022)	2012–2025, 2026–2031	3
Mueller et al (2024) (96)	Japan (HIC)	65 years	PCV21 vs PCV20	Markov (static)	CUA (QALY)	JPY (2023)	Lifetime	2
Rey-Ares et al (2024) (97)	Argentina (UMIC)	0–23 months	PCV20 vs PCV13 PCV20 vs PCV15	Markov (static)	CUA (QALY)	USD (2023)	10 years	3
Rozenbaum et al (2024) (98)	United States (HIC)	<2 years	PCV20 vs PCV13 PCV20 vs PCV15	Markov (static)	CUA (QALY)	USD (2022)	10 years	3
Shinjoh et al (2024) (99)	Japan (HIC)	birth cohort	PCV15 vs PCV13 PCV20 vs PCV13 PCV20 vs PCV15	Markov (static)	CUA (QALY)	JPY (2022)	10 years	2
Ta et al (2024) (100)	Germany (HIC)	<2 years	PCV20 vs PCV13 PCV20 vs PCV15	Markov (static)	CEA (LYG), CUA (QALY)	Euro (2022)	10 years	3
Yi et al (2024) (101)	United States (HIC)	≥65 years	PCV21 vs PCV20	Markov (static)	CUA (QALY)	USD (2023)	Lifetime	3

Abbreviations: CAD, Canadian dollar; CBA, cost-benefit analysis; CEA, cost-effectiveness analysis; CUA, cost-utility analysis; DALYs, Disability-adjusted life years; GBP, UK pounds; HIC, high-income countries; I$, International dollars; JPY, Japanese Yen; LIC, low-income countries; LYG, life year gained; LMIC, lower-middle-income countries; NR, not reported; NV, no vaccination; PCVs, pneumococcal conjugate vaccines; PHP, Philippine Pesos; QALY, Quality-adjusted life year; ROR, rate of return; SEK, Swedish Krona; THB, Thai Baht; UMIC, upper-middle-income countries; USD, United States dollar; YOLS, years of life saved.

Modeling was used in all studies. Most studies (*n* = 45) applied a static modeling, whereas dynamic modeling was undertaken in five studies (24;53;75;86;89). The remaining seven studies did not explicitly state their methods (51;59;61;67;87;90;91). PCV13 was the most frequently evaluated vaccine (*n* = 39), especially for children (*n* = 33), often compared to no vaccination and PCV10. The type of analyses included twenty-eight cost-per-QALY studies, fourteen cost-per-DALY studies, and five cost-per-LYs studies. Nine studies included both per-QALY and per-LYs analyses. One study applied rate of return and cost-per-QALY analyses (82). Twenty-four studies projected outcomes over a lifetime horizon (47–49;51;56;59;61;62;64;68;69;71;75;79–84;86;90;91;94;96;101), whereas one study did not report a time horizon (54). The remaining studies used time horizons between one year and fifty years. All studies applied discounting, except for one EE with a one-year time horizon (52). Thirty-one studies analyzed both societal and healthcare or payer perspectives (24;50;52;53;58;60;63;65–68;70;72;75–78;81;82;84;85;88–90;93;95–99;101). All studies explicitly reported on sensitivity analysis, except for one study (74). Almost half of the studies (*n* = 28) were funded by the industries (47;50;52;57;59;63;64;69;71;73;75–77;81;82;84–86;88;89;93;94;96–101). Additional information about the studies’ characteristics is presented in Supplementary File 4.

### Quality assessment

The quality scores of the included economic analyses ranged from 65 percent to 95 percent, with a mean score of 85 percent. The most recent studies (*n* = 30) demonstrated higher quality, particularly in the last five years (2020–2024), with a mean score of 86.43 percent. However, the studies most often failed to account for appropriate study design and lacked clear mentions of the model structural assumptions and validation methods. Identification of costs varied across studies and was not comprehensively reported in twenty-seven studies (24;48–51;57;58;62–66;68–70;72–74;76–79;81;83;87;88;95). Approximately half of the studies were scored partially on the generalizability item because it was discussed only briefly or lacked broader applicability. The additional item most poorly reported was ethical and distributional considerations. Details of the quality assessment for each individual study can be found in Supplementary File 5.

### Identification and categorization of intersectoral costs and benefits (ICBs)

Table 2 summarizes which ICBs were identified in the included studies for both children and adults. These costs and benefits were placed under the following sectors: patient and family, paid labor (productivity), nonpaid productivity (opportunity costs), other sectors (education, leisure, consumption), and unspecified sector for ecological effects (herd immunity, serotype replacement, cross protection, antimicrobial resistance), and equity. The most included ICBs were absence from work, followed by herd immunity, out-of-pocket and serotype replacement. The number and proportion of studies including ICBs are presented in Supplementary File 6 over the year of publication. Additional information on the ICBs captured in each individual study is presented in Supplementary File 7.

**Table 2.** Details on intersectoral costs and benefits identified in the selected studies per sector, age, and income level **^a^** Table 2. long description.

**Sectors &** **costs/benefits components**	High-income countries (HICs)		Low- and middle-income countries (LMICs)	
	Child (*N* = 19)	Adult (*N* = 11)	Child (*N* = 28)	Adult (*N* = 1)
	*n (%)*	*n (%)*	*n (%)*	*n (%)*
**Patient and family^b^**				
OOP caregiver	5 *(26)*	–	17 *(61)*	–
OOP patient	1 *(5)*	3 *(27)*	–	1 *(100)*
Relatives’ time	–	–	2 *(7)*	–
**Paid labor (productivity)^b^**				
Absence caregiver	17 *(89)*	1 *(9)*	16 *(57)*	1 *(100)*
Absence patient	10 *(53)*	9 *(82)*	2 *(7)*	–
Mortality	4 *(21)*	5 *(45)*	1 *(4)*	–
Disability	2 *(11)*	3 *(27)*	2 *(7)*	–
**Non-paid productivity^b^**				
Unpaid work	–	1 *(9)*	1 *(4)*	–
**Education^b^**				
Special education	3 *(16)*	–	5 *(18)*	–
**Leisure^b^**				
Leisure activities	–	–	2 *(7)*	–
**Consumption^b^**				
Future nonmedical consumption cost	–	1 *(9)*	–	–
**Unspecified^c^**				
Serotype replacement	7 *(37)*	2 *(18)*	13 *(46)*	–
Herd immunity	17 *(89)*	6 *(55)*	22 *(79)*	–
Cross protection	3 *(16)*	1 *(9)*	2 *(7)*	–
Antimicrobial resistance	–	1 *(9)*	–	–
Equity	–	1 *(9)*	–	–

a The studies by Wu et al. (63) and Chen et al. (24) were accounted for both HICs and LMICs countries. These studies included children from both income levels.

b Costs categories adapted from Drummond et al. (38).

c Benefits categories adapted from Jit et al. (2).

Abbreviations: *N*, total number of studies; *n*, Number of studies that captured one or more cost/benefit components in each sector; OOP, out-of-pocket.

#### Intersectoral costs and benefits among children

Among children-based studies (*n* = 45), patient and family costs were captured in twenty-two studies (49 percent) that could be placed under the following cost categories: out-of-pocket (OOP) costs for caregiver/family (*n* = 21) (24;46;48–50;54;56;58;60;65–67;69;70;72;76–78;84;87;95), OOP costs for patient (*n* = 1) (65), and relatives’ time (*n* = 2) (51;78). Paid labor (productivity) was the main sector assessed in the included studies (*n* = 33; 73 percent). Productivity costs were most frequently captured in terms of absence from work for caregiver (*n* = 31) (24;46–50;53–56;60;61;63–69;73;84–86;88;89;93;94;97–100), followed by absence from work for patients (*n* = 12) (47;48;53;64;82;84;88;89;93;94;98;99), mortality (*n* = 5) (53;67;84;88;94), and disability (*n* = 4) (65;80;86;98). Patients’ work loss as spill-over effect in the community was mainly assessed in the studies from HICs (*n* = 6) (53;88;89;93;98;99).

The education sector was captured in eight studies (18 percent) in terms of special education due to meningitis sequelae. Of these, seven studies assessed education cost alongside healthcare costs (47;56–58;61;62;79), whereas one study reported this cost for family (84). Unpaid work was considered in one study (2 percent) due to caregivers’ time loss and patients’ time loss (82). In this study, the caregivers’ time was equated to opportunity cost, whereas the patient’s time was equated to replacement cost. Furthermore, two studies (4 percent) captured leisure sector (80;82) in terms of value of lost leisure time for caregivers while taking care of sick children.

Thirty-seven studies (82 percent) captured community or health system externalities under the “unspecified” sector in terms of ecological effects (herd immunity, serotype replacement, cross protection). All thirty-seven studies included herd immunity (24;46–50;53;54;56–58;60;62–70;73;77;79;80;82;84–88;93;94;97–100). The majority of these studies (*n* = 28) included herd immunity in the main analysis (24;47–49;56–58;60;62–66;68;70;73;77;80;82;84;86–88;93;94;97;99;100). One or combined sources were used to account for herd immunity. Fifteen studies estimated herd immunity using surveillance data from United Kingdom (UK) (57;63;64;66;73;82;93;100), United States (USA) (47–49;56;63;84), The Netherlands (57;73), Wales (64;100), Finland (73;82), Germany (94), and Argentina (97). Ten studies used assumptions to account for herd immunity (24;62;68;70;77;80;86–88;99), three used vaccine coverage (48;65;82), three estimated herd immunity from literature (62;68;80), two implied a simple multiplier (58;60), and one study used guidance from expert panel (48).

We identified nineteen studies that accounted for serotype replacement. Fifteen studies considered serotype replacement in their main analysis (24;47–49;56–58;60;65;66;73;77;82;94;100). Here, the serotype replacement was estimated using surveillance data from the United Kingdom (57;63;66;73;77;82), Finland (73;77;82), USA (47;63), Germany (94), and The Netherlands (57). Six studies applied assumption (24;49;56;58;60;100), three considered vaccine coverage (48;58;65), and one study used guidance from expert panel (48). The remaining studies (*n* = 4) varied serotype replacement in sensitivity analysis (46;50;54;67). Cross-protection was considered in five studies (48;57;82;85;93). There were different scenarios in the assumptions between the studies. Cross-protection from serotype 6A/6B for PCV10 and PCV13 (48), from PCV10 against serotype 19A (57), from PCV10 for serotypes 19A or 6A (82), against serotype 6C for PCV13, 15-valent (PCV15) and 20-valent (PCV20), and 15C for PCV20 (85), and 6C and 15C for PCV20 versus PCV15 (93). Only one study (93) explicitly reported the level of cross-protection: 93.7 percent for 6C and 88.7 percent for 15C.

#### Intersectoral costs and benefits among adults

Among adult-based studies (*n* = 12), only four studies (33 percent) considered patient and family sector costs in terms of OOP costs incurred by patients (52;71;83;101). Meanwhile, all these studies captured the paid labor (productivity) sector, including patients’ costs in terms of work loss (absenteeism) (59;71;74;75;81;90–92;96), disease-related mortality (90–92;96;101), and disability (52;90;91). Furthermore, two studies estimated caregivers’ costs in terms of work loss due to caring for adult patients aged ≥65 years (75;83). Nonpaid productivity cost (unpaid work) was only estimated in one study (8 percent) (59) for both patients and caregivers in terms of replacement cost and opportunity cost, respectively. One study (74) accounted for consumption in terms of future non-medical costs (NMCs). These costs refer to expenditure beyond the healthcare sector, such as costs of housing in the added years. In this study, NMC was estimated by the tool Practical Application to Include future Disease costs (PAID) using Dutch Household Consumption surveys. PAID provides estimates for NMC by age.

Seven studies (58 percent) accounted for community or health system externalities under the unspecified sector in terms of ecological effects and equity. Herd immunity was considered in six studies due to the use of PCVs in children (52;59;71;90;92;101). Three studies estimated herd immunity in the main analysis using surveillance data from the United States (52), literature and vaccine coverage (71), and one applied assumptions and used observations from the multicountry studies (92). Meanwhile, two studies captured serotype replacement in their analyses (59;92). Of these, one study (92) measured serotype replacement in the main analysis using assumptions and observations from the multicountry studies. Cross-protection to serotype 6C was accounted for by one study (92). One study (81) considered antimicrobial resistance (AMR),18 percent across serotypes, of PCV15 versus no vaccination in the scenario analysis using the Swiss Federal Office of Public Health. Furthermore, two studies (90;91) addressed equity in terms of racial disparities (Black versus non-Black populations).

### Illustration of the possible impact of intersectoral costs and benefits

ICER was reported in all studies, except for one study (86). However, eleven studies (24;48;51;52;61;62;79;80;83;86;92) did not provide sufficient information to assess the impact of ICBs’ inclusion (e.g., ICER was presented only for the base-case). Within the remaining studies (*n* = 46), thirty compared the target vaccination strategy versus no vaccination, and twenty-three compared the target vaccination strategy versus the comparator vaccination strategy (head-to-head), with a total of seventy separate comparisons. Of these, seven studies have reported more than one comparison (versus comparator vaccination strategy and no vaccination). Some comparisons could have reported multiple ICERs. Detailed information is presented in Supplementary File 8.

In cases where the comparator was no vaccination, the median ICER with and without ecological effects and productivity loss is shown in Tables 3 and 4, respectively. The median ICER varied by outcome and country income level, with the smallest median ICERs observed in DALY- and LMIC-based studies. Nine studies reported that PCVs were dominant when ecological effects were included, whereas six studies reported that PCVs were dominant when productivity loss and/or patient & family costs were included. After excluding dominant analyses, the impact of including ecological effects on the median ICER is greater than that of productivity. The median QALY-based ICER reductions ranged from −3.25 percent (for patient/family costs and productivity loss) to −46.02 percent (in children with ecological effects). Studies that included ecological effects had more favorable median QALY-based ICERs in LMICs (−57.23 percent) than in HICs (−8.73 percent). Studies funded by industry and other sources (e.g., Bill & Melinda Gates Foundation) also supported the inclusion of ICBs. In terms of specific ecological effects, the inclusion of herd immunity reduced the median QALY-based ICER by −12.86 percent (Supplementary File 8). In head-to-head comparisons, sixteen studies reported that PCVs were dominant when ICBs were included (Supplementary File 8).

**Table 3.** Change in ICER when ecological effects are included in the economic evaluation of PCVs versus no vaccination, presented in US$ 2024 Table 3. long description.

ICER^/^^a^	Without ecological effects			With ecological effects				
	Median	1stQuartile	3rdQuartile	Median	1st Quartile	3rd Quartile	Percentage reduction in ICER with ecological effects (median)^/^^b^	Studies
***$/QALY (n = 11)***	$65,963	$36,912	$157,674	$48,761	$16,820	$85,768	−42.97	(47;56;63;68;72;82;83;91)
Children (*n* = 8)	$88,539	$40,888	$160,875	$58,996	$17,227	$86,041	−46.02	(47;56;63;68;83)
HICs (*n* = 5)	$96,656	$36,320	$160,875	$72,689	$37,708	$87,060	−8.73	(47;72;82;91)
LMICs (*n* = 6)	$41,939	$32,355	$77,772	$18,306	$13,316	$47,030	−57.23	(56;63;68;83)
Funded by industry (*n* = 7)	$44,030	$31,336	$113,915	$37,579	$14,194	$86,041	−29.71	(47;63;72;82;83)
***$/DALY (n = 8)***	$2,789	$187	$3,278	$2,104	$132	$3,205	−22.97	(46;49;54;66)
Children (*n* = 8)	$2,789	$187	$3,278	$2,104	$132	$3,205	−22.97	(46;49;54;66)
LMICs (*n* = 8)	$2,789	$187	$3,278	$2,104	$132	$3,205	−22.97	(46;49;54;66)
Funded by other sources (*n* = 8)	$2,789	$187	$3,278	$2,104	$132	$3,205	−22.97	(46;49;54;66)

a Cost-saving results are not included. Subgroups with fewer than five comparisons were not presented in this table. Each comparison could have reported multiple ICERs.

b The median of the percentage reduction of each ICER pair. It does not equal the actual ICER reduction with ecological effects over the ICER without ecological effects. Negative % indicates that ICERs were more favorable when ICBs were included.

Abbreviations: DALYs, Disability-adjusted life years; HIC, high-income countries; ICER, incremental cost-effectiveness ratio; LMIC, lower-middle-income countries; *n*, number of comparisons; QALY, Quality-adjusted life year; PCVs, pneumococcal conjugate vaccines.

**Table 4.** Change in ICER when productivity costs are included in the economic evaluation of PCVs versus no vaccination, presented in US$ 2024 Table 4. long description.

ICER^/^^a^	Without productivity			With productivity			Percentage reduction in ICER with productivity (median)^/^^b^	Studies
	Median	1stQuartile	3rdQuartile	Median	1st Quartile	3rd Quartile		
***$/QALY (n = 9)***	$61,426	$16,385	$92,681	$35,685	$14,322	$66,703	−12.59	(47;55;63;68;76;82;91)
Children (*n* = 6)	$76,334	$40,300	$92,681	$59,216	$35,685	$86,041	−11.45	(47;55;63;68)
HICs (*n* = 7)	$81,958	$14,211	$107,568	$40,909	$14,755	$81,207	−12.60	(47;55;63;76;82;91)
Funded by industry (*n* = 6)	$39,866	$14,211	$84,769	$37,634	$14,074	$81,207	−8.09	(47;63;76;82)
***$/LY (n = 5)***	$80,771	$15,734	$196,584	$52,834	$13,791	$137,251	−18.09	(53;55;63;76)

a Cost-saving results are not included. Subgroups with fewer than five comparisons were not presented in this table. Each comparison could have reported multiple ICERs.

b The median of the percentage reduction of each ICER pair. It does not equal the actual ICER reduction with ecological effects over the ICER without ecological effects. Negative % indicates that ICERs were more favorable when ICBs were included.

Abbreviations: HIC, high-income countries; ICER, incremental cost-effectiveness ratio; LY, life years; *n*, number of comparisons; QALY, Quality-adjusted life year; PCVs, pneumococcal conjugate vaccines.

## Discussion

To the best of our knowledge, this review is the first to explore the ICBs and to illustrate the impact of ICBs on the reported ICER in full EEs of PCVs across all age groups worldwide. The measurement of ICBs has increased over the years, in line with the increase in full EEs of PCVs. Furthermore, we found that understanding the epidemiological data of PDs and the availability of real-world data reshaped vaccine policy decisions (102). This trend has been observed in the studies (*n* = 17; 30 percent) that were published in 2023–2024. These studies focused on higher valent vaccines (PCV15, PCV20, PCV21).

Drummond’s framework (38) does not comprehensively cover the full value of vaccination, as argued for by the vaccine assessment framework (2). It does not include ecological effects and equity considerations. The basis for ICBs is externalities or spillover impacts (6). While intersectoral costs are defined as costs that arise in sectors outside of the one directly involved, spillover effects refer to how an intervention in one sector can have an impact on another sector. Public health interventions (e.g., PCVs) provide spillover health improvement in unvaccinated community members (1;2). Such improvements lead to economic growth through different ways (e.g., improve school attainment) (2). It should be noted that the impact has to be counted, either as a cost or a benefit to avoid double-counting in EEs (103). Therefore, this review used the term ICBs using both Drummond’s framework (38) and the vaccine assessment framework by Jit et al. (2).

This review reveals that cost bearers varied according to patients’ age group. Studies that focused on children predominantly included ICBs incurred by the family/caregivers (e.g., absenteeism from work for the caregiver). Studies that focused on adults are, in contrast, primarily considered ICBs incurred by the patient (e.g., absenteeism from work for the patient). There remain issues that warrant discussion. Most health economic evaluations of childhood vaccination may neglect the long-term effects on a child’s productivity or future loss (18). Additionally, Sevilla et al. argued that patients’ work loss as a spillover effect on their caregivers should be included in adult models to cover the full value of vaccination (104). Consequently, these studies are likely to underestimate the economic impacts on the working-age adults. This review also demonstrates that absence from work was the most frequently considered ICBs across the included studies, followed by herd immunity, out-of-pocket, and serotype replacement. However, we found that studies often consider a limited range of ICBs under the societal perspective. Sittimart et al. argued that the way the societal perspective is conceptualized and implemented in practice can vary across studies (5). This review showed that studies that included productivity and patient/family costs showed favorable median ICERs.

We identified several gaps in the included studies that could serve as policy implications and recommendations for future research. First, Sustainable Development Goal 3 (SDG 3) emphasizes preventing infectious diseases to attain good health and well-being, such as through investment in immunization programs (105). As of May 2026, 177 countries globally have implemented PCV into their national immunization plans; eight are still considering implementation; eight have yet to make a decision; and one country has suspended the program. Of these, fifty-four countries are eligible for the Global Alliance for Vaccines and Immunization (Gavi) financial support (106). In this review, however, a small number of EEs were conducted in eligible countries receiving Gavi support, particularly in lower-middle-income and lower-income countries. While discontinuation of Gavi support is linked with country income level (21), several countries are expected to transition from this support to self-financing. Thus, more EEs of PCVs are needed in these countries to expand or support immunization programs.

Second, the inclusion of herd immunity and serotype replacement is driven by the availability of epidemiological data and/or model assumptions, particularly in LMICs (21). We observed a favorable median QALY-based ICER when including herd immunity. Conversely, the presence of serotype replacement (46;94) led to an increase in ICER. Wang et al. (21) argued that considering herd immunity without accounting for serotype replacement might overestimate the indirect effect of PCVs. Hence, the impact of herd immunity should be interpreted with caution. Furthermore, a recent study in LMICs revealed that studies that included herd immunity consistently had more favorable ICERs (41).

Third, a small number of EEs were identified that captured cross-protection, AMR, and equity considerations, making deriving a robust conclusion difficult. This could be explained by the fact that we excluded the studies that focused on healthcare or payer perspective. However, the authors of these studies reported that accounting for the reduction in total costs of AMR treatment (81) and cross-protection (85;93) slightly improved the cost-effectiveness results.

Fourth, in contrast to the static model, the dynamic model is able to account for disease transmission in a population (41;107). Hence, the dynamic model may have the potential to better account for the indirect effects and cost-effectiveness results (107). This review did not provide evidence for ICER impact in the dynamic versus static model because the included studies (24;53;75;86;89) with the dynamic model did not provide sufficient data to extract the impact of indirect effects. Additionally, there is no gold standard method to account for indirect effects (like herd immunity and serotype replacement) (41). This can be best seen in the included EEs with static models. These studies applied different methods to account for indirect effects, such as a simple multiplier, assumption, fixed percentage, and surveillance data (see Supplementary File 7). Wang et al. (21) argued that, local epidemiological data is often lacking, particularly in countries with no prior vaccination experience of PCVs. However, high-quality surveillance data is considered a reliable source to quantify for indirect effects compared to other methods used, particularly when using a static model (107).

Fifth, even though the quality of studies has improved over the years, we found that approximately half of the studies did not comprehensively report costs per sector, which likely influences the interpretation of the results. Furthermore, heterogeneity in the methods used (e.g., surveillance data, assumptions) to account for indirect effects poses challenges for comparability across studies. Hence, future studies should transparently justify or explain the guidance for reporting indirect effects, not just on model design (static versus dynamic) (108). This can also be seen in the quality assessment of the items on “study design” and “model structure,” where most of the studies were partially scored. Moreover, ethical and distributional considerations were poorly reported. It has also been argued that the role of economic evaluation of vaccines should be extended to ensure equitable distribution of health (20). However, we found that only two studies provided some evidence for the presence of racial inequalities (90;91). In these studies, researchers found that the black or African American population in the United States benefited more from the implementation of PCVs than the white population, as the ICER was dominant (Supplementary File 8). Therefore, subgroup analyses are recommended in future research, where applicable, to support the implementation of successful PCV policies and reduce inequality within and among countries (105). The identified gaps in studies’ quality may limit the transferability of our findings.

This study has several strengths and limitations. A comprehensive search strategy based on previous systematic reviews was implemented, including seven bibliographic databases. No restrictions were placed on the country or age. The citation search identified only one additional study, thereby further enhancing the reliability and validity of the search strategy. However, our review was limited to original studies published in English, which may have restricted the comprehensiveness of the review.

Identified ICBs were placed under predefined classification schemes, which can be adapted and expanded in future research. However, the included studies were heterogeneous in their consideration of ICB consequences. This observation aligns with a recent study by Schnitzler et al. (16), who acknowledged the methodological challenges when assessing ICBs in economic evaluations, particularly in the context of public health interventions. Accordingly, our findings would be interpreted as the magnitude or potential direction of impacts, rather than serving as a basis for direct comparisons across the studies. This review discussed some important policy implications related to the Sustainable Development Goals, such as the prevention of infectious diseases and racial inequality. Finally, we were unable to draw a robust conclusion about the magnitude of impact of ICBs’ inclusion, especially by funding sources, specific cost/benefit components, and when comparing children to the adult population, because dominant ICERs were excluded from the analysis.

## Conclusion

The limited perspective of EEs in the field of pneumococcal conjugate vaccines poses the risk of omitting important costs and benefits, which likely influences the vaccine policy decision. We observed a reduction in ICERs with ICBs. However, excluding dominant analyses may have led to an underrepresentation of intersectoral impacts. Although ICBs have been used more in EEs in the last 5 years, some ICBs are often overlooked in EEs studies of PCVs, such as education, nonpaid productivity, and equity considerations. Standardization of EEs of PCVs is recommended in future research, for example, in WHO Guide (108). However, the outcome of this systematic review provides a comprehensive framework of costs and benefits that could be used in future research.

## Supporting information

10.1017/S0266462326103912.sm001 Almomani et al. supplementary material Almomani et al. supplementary material

## Data Availability

The data used in this study are available from the corresponding author upon reasonable request.

## Supplementary material

The supplementary material for this article can be found at http://doi.org/10.1017/S0266462326103912.

## Long descriptions

### Figure 1. Long description

The flowchart is divided into three vertical phases: Identification, Screening, and Included.

1. Identification Phase (Top Section):

- Left Track (Databases and registers): Records identified from Databases (n = 7), M E D L I N E (Ovid) (n = 623), Embase (Ovid) (n = 899), Web of Science (Core Collection) (n = 960), C I N A H L (E B S C O) (n = 129), Psyc I N F O (E B S C O) (n = 9), Econ Lit (E B S C O) (n = 5), and N H S E E D (n = 49). An arrow points right to ‘Records removed before screening: Duplicate records removed (n = 1,290)'.

- Right Track (Other methods): Records identified from Citation searching (n = 1,889).

2. Screening Phase (Middle Section):

- Left Track: ‘Records screened (n = 1,384)' leads to ‘Records excluded (n = 1,259)'. The remaining ‘Reports sought for retrieval (n = 125)' leads to ‘Reports not retrieved (n = 0)'. Then, ‘Reports assessed for eligibility (n = 125)' leads to ‘Reports excluded: Not adopted or included a societal perspective (n = 65) and Not relevant comparator (n = 4)'.

- Right Track: ‘Reports sought for retrieval (n = 1,889)' leads to ‘Reports not retrieved (n = 0)'. Then, ‘Reports assessed for eligibility (n = 1,889)' leads to ‘Reports excluded: Not met the eligibility criteria (n = 1,832) and Duplicates eligible records retrieved from original databases (n = 55)'.

3. Included Phase (Bottom Section):

- Both tracks converge into a final box: ‘Studies included in review (n = 57): Databases (n = 56) and Citation searching (n = 1)'.

Navigate back to Figure 1..

### Table 1. Long description

The table is organized into nine columns: Study (year), Country (income level), Population, Comparison, Type of approach, Type of analysis (Outcome), Currency (year), Time horizon, and Discount rate (%).

Key entries include:

* Kim et al (2010): Gambia (L I C), birth cohorts, P C V 9/10/13 vs N V, Markov (static), C E A (D A L Y), U S D (2005), 5 years, 3%.

* Rozenbaum et al (2010): Netherlands (H I C), newborn cohorts, P C V 10/13 vs N V, Decision tree (static), C U A (Q A L Y), Euro, 5 years or Lifetime, 4% for cost and 1.5% for outcome.

* Castaneda-Orjuela et al (2012): Colombia (U M I C), children < 1 year, P C V 10 vs N V or P C V 13 vs P C V 10, Markov, C E A (L Y G), U S D (2009), Lifetime, 3%.

* Wu et al (2012): Taiwan (H I C), birth cohort, P C V 13 vs N V, Transmission (Dynamic), C E A (L Y G), U S D (2009), 10 years, 3%.

* Chen et al (2019): Global, 30 birth cohorts, P C V 13 vs N V, Ecological and Decision tree (dynamic), C E A (D A L Y), I $ (2015), 30 years, 3%.

* Altawalbeh et al (2024): United States (H I C), age 50 plus, P C V 21 vs N V or P C V 21 vs P C V 20, Markov, C U A (Q A L Y), U S D (2019), Lifetime, 3%.

* Shinjoh et al (2024): Japan (H I C), birth cohort, P C V 15/20 vs P C V 13 or P C V 20 vs P C V 15, Markov (static), C U A (Q A L Y), J P Y (2022), 10 years, 2%.

The table tracks the evolution of vaccine comparisons from early P C V 7 and P C V 9 studies to recent evaluations of P C V 15, P C V 20, and P C V 21.

Navigate back to Table 1..

### Table 2. Long description

The table is structured with five columns. The first column lists Sectors and costs/benefits components. The next four columns are grouped under High-income countries (H I C s) and Low- and middle-income countries (L M I C s), each subdivided into Child and Adult categories with their respective study counts (N) and percentages (n %).

Key data points include:

* Patient and family: O O P caregiver costs are reported in 26% of H I C child studies and 61% of L M I C child studies. O O P patient costs are highest in the L M I C adult category at 100% (n=1).

* Paid labor (productivity): Absence of caregiver is a major component, appearing in 89% of H I C child studies and 57% of L M I C child studies. Absence of patient is reported in 82% of H I C adult studies.

* Education: Special education costs are noted in 16% of H I C child studies and 18% of L M I C child studies.

* Unspecified benefits: Herd immunity is highly prevalent, cited in 89% of H I C child studies and 79% of L M I C child studies. Serotype replacement is noted in 37% of H I C child studies and 46% of L M I C child studies.

* Other categories like Non-paid productivity, Leisure, and Consumption show minimal reporting across all groups, often with zero or single-digit percentages.

Navigate back to Table 2..

### Table 3. Long description

The table is structured with nine columns. The first column identifies the I C E R category and subgroup. Columns 2 through 4 show data without ecological effects (Median, 1st Quartile, 3rd Quartile). Columns 5 through 7 show data with ecological effects (Median, 1st Quartile, 3rd Quartile). Column 8 shows the percentage reduction in I C E R with ecological effects (median), and Column 9 lists the study references.

Key data points for dollars per Q A L Y (n = 11):

* Overall: Median I C E R drops from $65,963 to $48,761 (-42.97 percent).

* Children (n = 8): Median I C E R drops from $88,539 to $58,996 (-46.02 percent).

* H I C s (n = 5): Median I C E R drops from $96,656 to $72,689 (-8.73 percent).

* L M I C s (n = 6): Median I C E R drops from $41,939 to $18,306 (-57.23 percent).

* Funded by industry (n = 7): Median I C E R drops from $44,030 to $37,579 (-29.71 percent).

Key data points for dollars per D A L Y (n = 8):

* All subgroups (Children, L M I C s, and Funded by other sources) show identical values: Median I C E R drops from $2,789 to $2,104, representing a -22.97 percent reduction. The 1st Quartile is $187 without and $132 with ecological effects; the 3rd Quartile is $3,278 without and $3,205 with ecological effects.

Navigate back to Table 3..

### Table 4. Long description

The table is organized into nine columns. The first column lists the I C E R metric or subgroup. Columns two through four show the Median, 1st Quartile, and 3rd Quartile for results Without productivity. Columns five through seven show the Median, 1st Quartile, and 3rd Quartile for results With productivity. Column eight shows the Percentage reduction in I C E R with productivity (median), and column nine lists the study references.

* Row 1: Dollars per Q A L Y (n = 9). Without productivity: Median $61,426, 1st Quartile $16,385, 3rd Quartile $92,681. With productivity: Median $35,685, 1st Quartile $14,322, 3rd Quartile $66,703. Percentage reduction: -12.59.

* Row 2: Children (n = 6). Without productivity: Median $76,334, 1st Quartile $40,300, 3rd Quartile $92,681. With productivity: Median $59,216, 1st Quartile $35,685, 3rd Quartile $86,041. Percentage reduction: -11.45.

* Row 3: H I C s (n = 7). Without productivity: Median $81,958, 1st Quartile $14,211, 3rd Quartile $107,568. With productivity: Median $40,909, 1st Quartile $14,755, 3rd Quartile $81,207. Percentage reduction: -12.60.

* Row 4: Funded by industry (n = 6). Without productivity: Median $39,866, 1st Quartile $14,211, 3rd Quartile $84,769. With productivity: Median $37,634, 1st Quartile $14,074, 3rd Quartile $81,207. Percentage reduction: -8.09.

* Row 5: Dollars per L Y (n = 5). Without productivity: Median $80,771, 1st Quartile $15,734, 3rd Quartile $196,584. With productivity: Median $52,834, 1st Quartile $13,791, 3rd Quartile $137,251. Percentage reduction: -18.09.

Navigate back to Table 4..
