# Supplementary material for: Consideration of intersectoral costs and benefits in health economic evaluations of pneumococcal conjugate vaccines: a systematic review
Source: Int J Technol Assess Health Care. 2026 Jul 17;42(1):e59. doi: 10.1017/S0266462326103912 (PMC13434861; doi:10.1017/S0266462326103912)
Supplement: Supplementary file 1 [file S0266462326103912sup001.zip › Supplemental_File_5.pdf]

Supplemental file 5: Quality assessment of modelling studies

	Study population	Competing alternatives	Research question	Economic study design	Model description	Time horizon	Study perspective	Costs identification	Costs measurement	Costs valuation	Outcomes identification	Outcomes measurement	Outcomes valuation	Incremental CE analysis	Discounting	Uncertainty analysis	Conclusions	Generalizability	conflict of interest	Ethical issues	Score (%)
Kim 2010 (46)	Green	Green	Yellow	Yellow	Yellow	Yellow	Green	Green	Green	Green	Green	Green	Green	Green	Yellow	Green	Green	Green	Green	Yellow	85
Rozenbaum 2010 (47)	Green	Green	Green	Yellow	Yellow	Green	Green	Green	Yellow	Green	Green	Yellow	Yellow	Green	Green	Green	Yellow	Red	Green	Yellow	78
Nakamura 2011 (48)	Green	Green	Yellow	Yellow	Yellow	Green	Green	Yellow	Green	Green	Green	Green	Green	Green	Green	Yellow	Green	Yellow	Green	Yellow	83
Tasslimi 2011 (49)	Green	Green	Yellow	Yellow	Green	Green	Green	Yellow	Green	Green	Green	Green	Green	Yellow	Green	Yellow	Green	Yellow	Green	Yellow	83
Uruena 2011 (50)	Green	Green	Green	Yellow	Yellow	Yellow	Green	Yellow	Green	Green	Green	Yellow	Green	Green	Green	Yellow	Green	Yellow	Green	Red	78
Castaneda-Orjuela 2012 (51)	Green	Green	Green	Green	Yellow	Green	Yellow	Yellow	Yellow	Green	Green	Yellow	Yellow	Green	Yellow	Green	Green	Green	Green	Yellow	80
Kuhlmann 2012 (52)	Green	Green	Green	Yellow	Yellow	Yellow	Green	Green	Green	Green	Green	Green	Green	Green	Green	Green	Green	Yellow	Green	Red	85
Wu et al 2012 (53)	Yellow	Green	Green	Green	Green	Yellow	Green	Green	Green	Green	Green	Green	Green	Green	Green	Green	Green	Yellow	Green	Red	88
Ayieko 2013 (54)	Green	Green	Green	Yellow	Yellow	Red	Green	Green	Green	Green	Yellow	Green	Green	Green	Green	Yellow	Green	Yellow	Green	Red	78
Hoshi 2013 (55)	Green	Green	Yellow	Yellow	Yellow	Yellow	Green	Green	Green	Green	Green	Yellow	Yellow	Green	Green	Green	Green	Green	Green	Yellow	83
Kulpeng 2013 (56)	Yellow	Green	Yellow	Yellow	Yellow	Green	Green	Green	Green	Green	Green	Green	Green	Green	Green	Green	Green	Yellow	Green	Red	83
Vemer 2014 (57)	Green	Green	Yellow	Yellow	Yellow	Yellow	Green	Yellow	Green	Green	Green	Yellow	Red	Green	Green	Green	Green	Yellow	Green	Yellow	75
Kieninger 2015 (58)	Green	Green	Green	Yellow	Yellow	Yellow	Green	Yellow	Green	Green	Green	Green	Green	Green	Green	Green	Green	Yellow	Green	Red	83
Mangen 2015 (59)	Green	Green	Yellow	Green	Yellow	Green	Green	Green	Green	Green	Green	Green	Green	Green	Green	Yellow	Yellow	Yellow	Green	Yellow	83
Vucina 2015 (60)	Green	Green	Green	Yellow	Yellow	Yellow	Green	Green	Green	Green	Green	Green	Green	Green	Green	Yellow	Green	Yellow	Green	Red	83
Maurer 2016 (61)	Green	Green	Green	Green	Yellow	Green	Yellow	Green	Yellow	Green	Green	Yellow	Green	Green	Green	Green	Green	Green	Green	Yellow	88
Mo 2016 (62)	Green	Green	Yellow	Yellow	Yellow	Green	Green	Yellow	Green	Green	Green	Yellow	Green	Green	Green	Green	Green	Yellow	Green	Yellow	83
Wu 2016 (63)	Green	Green	Green	Yellow	Yellow	Yellow	Green	Yellow	Green	Green	Green	Green	Green	Green	Green	Green	Green	Green	Green	Red	85
Gouveia 2017 (64)	Green	Green	Green	Yellow	Yellow	Green	Green	Yellow	Green	Green	Green	Red	Yellow	Green	Green	Yellow	Yellow	Yellow	Green	Yellow	75
Kuhlmann 2017 (65)	Green	Green	Green	Yellow	Yellow	Green	Green	Yellow	Green	Green	Green	Green	Yellow	Green	Green	Green	Green	Green	Red	Red	80
Sundaram 2017 (66)	Green	Green	Green	Yellow	Yellow	Green	Green	Yellow	Green	Green	Green	Green	Green	Green	Green	Yellow	Green	Yellow	Green	Red	83
Lara 2018 (67)	Green	Green	Green	Green	Yellow	Yellow	Green	Green	Green	Green	Green	Green	Green	Green	Green	Green	Green	Yellow	Green	Red	88
Zhou 2018 (68)	Green	Green	Green	Yellow	Yellow	Green	Green	Yellow	Green	Green	Green	Green	Green	Green	Green	Green	Green	Green	Green	Red	88
Chen 2019 (69)	Green	Green	Green	Green	Yellow	Green	Yellow	Yellow	Green	Green	Green	Green	Green	Green	Yellow	Yellow	Green	Yellow	Green	Yellow	83
Dilokthornsakul 2019 (70)	Green	Green	Green	Yellow	Green	Green	Green	Yellow	Green	Green	Green	Green	Yellow	Green	Green	Green	Green	Green	Yellow	Yellow	88

Ezoji 2019 (71)	yes	yes	yes	Partial	No	Partial	Partial	Partial	yes	yes	yes	Partial	Partial	yes	Partial	Partial	yes	yes	yes	No	70
Gouveia 2019 (72)	yes	yes	yes	Partial	yes	yes	yes	yes	yes	yes	yes	yes	yes	yes	Partial	yes	yes	No	yes	Partial	88
Idris 2020 (73)	yes	yes	yes	Partial	Partial	Partial	yes	Partial	yes	yes	yes	yes	yes	yes	yes	yes	yes	yes	yes	No	85
Shafie 2020 (74)	Partial	yes	yes	Partial	Partial	Partial	yes	Partial	Partial	yes	yes	yes	yes	yes	yes	yes	yes	yes	yes	Partial	83
de Vries 2021 (75)	yes	yes	Partial	Partial	No	Partial	yes	Partial	yes	yes	yes	Partial	Partial	No	yes	No	yes	Partial	yes	Partial	65
Igarashi 2021 (76)	yes	yes	yes	yes	Partial	yes	yes	yes	yes	yes	yes	yes	yes	yes	yes	yes	yes	Partial	yes	No	90
Pecenka 2021 (77)	yes	yes	yes	Partial	Partial	Partial	yes	Partial	yes	yes	yes	yes	yes	yes	yes	yes	yes	Partial	yes	Partial	85
Perdrizet 2021 (78)	yes	yes	yes	Partial	Partial	Partial	yes	Partial	yes	yes	yes	yes	yes	yes	yes	yes	Partial	Partial	yes	Partial	83
Suwantika 2021 (79)	yes	yes	Partial	Partial	Partial	Partial	yes	Partial	yes	yes	yes	Partial	Partial	yes	Partial	Partial	yes	No	yes	No	68
Wang 2021 (80)	yes	yes	yes	Partial	Partial	yes	yes	Partial	Partial	yes	yes	yes	yes	yes	Partial	yes	yes	yes	yes	Partial	85
Chaiyakunapruk 2022 (81)	yes	yes	yes	Partial	Partial	yes	yes	yes	Partial	yes	yes	yes	yes	yes	yes	yes	yes	No	yes	Partial	85
Deb 2022 (82)	yes	yes	yes	Partial	Partial	yes	yes	Partial	yes	yes	yes	yes	Partial	yes	yes	yes	yes	Partial	yes	No	83
Sevilla 2022 (83)	yes	yes	yes	Partial	Partial	yes	yes	yes	Partial	yes	yes	yes	yes	yes	yes	yes	yes	No	yes	Partial	85
Guo 2023 (84)	yes	yes	yes	Partial	Partial	yes	yes	Partial	Partial	yes	yes	yes	yes	yes	yes	yes	yes	Partial	yes	Partial	85
Huang 2023 (85)	Partial	yes	yes	Partial	Partial	yes	yes	yes	yes	yes	yes	yes	yes	yes	yes	yes	yes	yes	yes	Partial	90
Lytle 2023 (86)	yes	yes	yes	Partial	Partial	yes	yes	yes	yes	yes	yes	yes	yes	yes	yes	yes	yes	Partial	yes	No	88
Ordonez 2023 (87)	yes	yes	yes	yes	Partial	yes	yes	yes	yes	yes	yes	Partial	yes	yes	yes	yes	yes	yes	yes	Partial	93
Prasad 2023 (88)	yes	yes	yes	Partial	Partial	Partial	yes	Partial	Partial	yes	yes	yes	yes	yes	yes	Partial	yes	Partial	yes	Partial	80
Tajima 2023 (89)	yes	yes	yes	Partial	Partial	yes	yes	Partial	yes	yes	yes	yes	yes	yes	yes	yes	yes	yes	yes	No	88
Wilson 2023 (90)	Partial	yes	yes	yes	Partial	Partial	yes	yes	yes	yes	yes	yes	yes	yes	yes	yes	yes	yes	yes	No	88
Altawalbeh 2024 (91)	yes	yes	yes	Partial	Partial	yes	yes	yes	yes	yes	yes	Partial	Partial	yes	yes	yes	yes	yes	yes	yes	90
Altawalbeh 2024 (92)	yes	yes	yes	yes	Partial	yes	yes	Partial	yes	yes	Partial	yes	yes	yes	yes	yes	yes	Partial	yes	yes	90
de Boer 2024 (93)	yes	yes	yes	Partial	Partial	yes	yes	yes	yes	yes	yes	Partial	Partial	yes	yes	yes	yes	yes	yes	Partial	88
Fridh 2024 (94)	yes	yes	yes	Partial	Partial	yes	yes	yes	yes	yes	yes	yes	yes	yes	yes	yes	yes	yes	yes	Partial	93
Huang 2024 (95)	yes	yes	yes	Partial	Partial	yes	yes	yes	yes	yes	yes	yes	yes	yes	yes	yes	yes	yes	yes	Partial	93
Ibrahim 2024 (96)	yes	yes	yes	Partial	Partial	Partial	yes	Partial	yes	yes	yes	Partial	Partial	yes	yes	yes	yes	yes	yes	Partial	83
Mueller 2024 (97)	yes	yes	yes	Partial	Partial	yes	yes	yes	yes	yes	yes	yes	yes	yes	yes	yes	yes	yes	yes	Partial	93
Rey-Ares 2024 (98)	yes	yes	yes	yes	Partial	yes	yes	yes	yes	yes	yes	yes	yes	yes	yes	yes	yes	yes	yes	Partial	95
Rozenbaum 2024 (99)	yes	yes	yes	yes	Partial	Partial	yes	yes	yes	yes	yes	yes	yes	yes	yes	yes	yes	yes	yes	Partial	93
Shinjoh 2024 (100)	yes	yes	yes	Partial	Partial	Partial	yes	yes	yes	yes	yes	yes	yes	yes	yes	yes	yes	Partial	yes	Partial	88
Ta 2024 (101)	yes	yes	yes	Partial	Partial	Partial	yes	yes	yes	yes	yes	yes	yes	yes	yes	yes	yes	yes	yes	Partial	90
Yi 2024 (102)	yes	yes	yes	Partial	yes	yes	yes	yes	Partial	yes	yes	Partial	yes	yes	yes	yes	yes	Partial	yes	Partial	88

yes Partial No
