# Supplementary material for: Consideration of intersectoral costs and benefits in health economic evaluations of pneumococcal conjugate vaccines: a systematic review
Source: Int J Technol Assess Health Care. 2026 Jul 17;42(1):e59. doi: 10.1017/S0266462326103912 (PMC13434861; doi:10.1017/S0266462326103912)
Supplement: Supplementary file 1 [file S0266462326103912sup001.zip › Supplemental_File_7.pdf]

Supplemental file 7: Intersectoral costs and benefits captured in each individual study

	OOP caregiver	OOP patient	Relatives' time	Absence caregiver	Absence patient	Disability	Mortality	Unpaid work	Special education	leisure	Consumption	Serotype replacement	Herd immunity	Cross protection	AMR	Equity
**Children**																
**Multiple country**																
Nakamura 2011 (48)	■			■	■							■	■	■		
Tasslimi 2011 (49)	■			■								■	■			
Wu 2016 (63)				■									■			
Chen 2019 (69)	■			■								■	■			
**HIC**																
Rozenbaum 2010 (47)				■	■				■			■	■			
Wu 2012 (53)				■	■		■						■			
Hoshi 2013 (55)				■												
Vemer 2014 (57)									■			■	■	■		
Vucina 2015 (60)	■			■								■	■			
Kuhlmann 2017 (65)	■	■		■		■						■	■			
Gouveia 2017 (64)				■	■								■			
Huang 2023 (85)	■			■	■		■		■				■			
Lytle 2023 (86)				■									■	■		
Prasad 2023 (88)	■												■			
Tajima 2023 (89)				■	■		■						■			
Wilson 2023 (90)				■	■											
Fridh 2024 (94)				■	■								■	■		
Huang 2024 (95)				■	■		■					■	■			
Rozenbaum 2024 (99)				■	■	■							■			
Shinjoh 2024 (100)				■	■								■			
Ta 2024 (101)				■								■	■			
**UMIC**																

	OOP caregiver	OOP patient	Relatives' time	Absence caregiver	Absence patient	Disability	Mortality	Unpaid work	Special education	leisure	Consumption	Serotype replacement	Herd immunity	Cross protection	AMR	Equity
Uruena 2011 (50)	■			■								■	■			
Castaneda-Orjuela 2012 (51)			■													
Kulpeng 2013 (56)	■			■					■			■	■			
Kieninger 2015 (58)	■								■			■	■			
Maurer 2016 (61)				■					■							
Mo 2016 (62)									■				■			
Sundaram 2017 (66)	■			■								■	■			
Lara 2018 (67)	■			■			■					■	■			
Zhou 2018 (68)				■									■			
Dilokthornsakul 2019 (70)	■			■									■			
Ezoji 2019 (71)	■												■			
Shafie 2020 (74)				■								■	■			
Suwantika 2021 (79)	■		■													
Wang 2021 (80)									■				■			
Chaiyakunapruk 2022 (81)						■				■			■			
Ordonez 2023 (87)				■		■							■			
Rey-Ares 2024 (98)				■									■			
**LMIC**																
Ayieko 2013 (54)	■			■								■	■			
Idris 2020 (73)	■															
Perdrizet 2021 (78)	■											■	■			
Sevilla 2022 (83)					■			■		■		■	■	■		
Ibrahim 2024 (96)	■															
**LIC**																
Kim 2010 (46)	■			■								■	■			
Pecenka 2021 (77)	■															
Total	***21***	***1***	***2***	***31***	***12***	***4***	***5***	***2***	***8***	***2***		***19***	***37***	***5***	***0***	***0***

	OOP caregiver	OOP patient	Relatives' time	Absence caregiver	Absence patient	Disability	Mortality	Unpaid work	Special education	leisure	Consumption	Serotype replacement	Herd immunity	Cross protection	AMR	Equity
**Adults**																
**HIC**																
Kuhlmann 2012 (52)		■				■							■			
Mangen 2015 (59)					■			■				■	■			
Gouveia 2019 (72)		■			■								■			
de Vries 2021 (75)					■						■					
Igarashi 2021 (76)				■	■											
Deb 2022 (82)					■										■	
Altawalbeh 2024 (91)					■	■	■						■			■
Altawalbeh 2024 (92)					■	■	■									■
de Boer 2024 (93)					■		■					■	■	■		
Mueller 2024 (97)					■		■									
Yi 2024 (102)		■					■						■			
**UMIC**																
Guo 2023 (84)		■		■												
Total	*0*	*4*	*0*	*2*	*8*	*2*	*4*	*1*	*0*	*0*	*1*	*2*	*6*	*1*	*1*	*1*

Abbreviations: AMR, antimicrobial resistance; HIC, high-income countries; LIC, low-income countries; LMIC, lower-middle-income countries; OOP, out-of-pocket; UMIC, upper-middle-income countries
