# Supplementary material for: Consideration of intersectoral costs and benefits in health economic evaluations of pneumococcal conjugate vaccines: a systematic review
Source: Int J Technol Assess Health Care. 2026 Jul 17;42(1):e59. doi: 10.1017/S0266462326103912 (PMC13434861; doi:10.1017/S0266462326103912)
Supplement: Supplementary file 1 [file S0266462326103912sup001.zip › Supplemental_File_8.pdf]

Supplemental file 8: Impact of intersectoral costs and benefits per individual study

Comparison	Age	Country (income level)	Year of evaluation	Main findings *[Adjusted ICER; impact on ICER %]* [a]
**PCV9 vs NV**	Child	Gambia (LIC) (46)	2005	**1. Main results:**<br>1.1. Societal (without indirect effects): $670/DALY. *[$3,278/DALY, Base case]*<br>**2. Scenario or sensitivity analysis results:**<br>2.1. With Serotype replacement (by 25%): $1,170/DALY. *[$5,725/DALY; 74.65%].*<br>2.2. With herd immunity (Assumed incidence decreased by 32%, 32%, 8%, and 18% for individuals aged 5-19 years, 20-39 years, 40-64 years, and 65 years and older): $430/DALY. *[$2,104/DALY; -35.81%].*<br>2.3. With indirect effects (Serotype replacement (increased incidence of non-primary endpoint pneumonia among vaccinated, and Herd immunity (assumed incidence decrease by 32%, 32%, 8%, and 18% for individuals aged 5-19 years, 20-39 years, 40-64 years, and 65 years and older): $550/DALY. *[$2691/DALY; -17.91%].*
**PCV10 vs NV**	Child	Gambia (LIC) (46)	2005	**1. Main results:**<br>1.1. Societal (without indirect effects): $670/DALY. *[$3,278/DALY, Base case]*<br>**2. Scenario or sensitivity analysis results:**<br>2.1. With Serotype replacement (by 25%): $1,170/DALY. *[$5,725/DALY; 74.65%].*<br>2.2. With herd immunity (Assumed incidence decreased by 32%, 32%, 8%, and 18% for individuals aged 5-19 years, 20-39 years, 40-64 years, and 65 years and older): $430/DALY. *[$2,104/DALY; -35.81%].*<br>2.3. With indirect effects (Serotype replacement (increased incidence of non-primary endpoint pneumonia among vaccinated, and Herd immunity (assumed incidence decrease by 32%, 32%, 8%, and 18% for individuals aged 5-19 years, 20-39 years, 40-64 years, and 65 years and older): $550/DALY. *[$2691/DALY; -17.91%].*
	Child	Kenya (LMIC) (54)	2010	**1. Main results:**<br>1.1. Societal and no indirect effects: KES4,675/DALY. [$191/DALY; Base case]<br>**2. Scenario or sensitivity analysis results:**<br>2.1. Societal and indirect effects in the entire population: KES2,535/DALY. *[$104/DALY; -45.55%]*
	Child	Egypt (LMIC) (83)	2016	**1. Main results:**<br>1.1. Payer (Indirect effects of IPD allowed for all ages. Indirect effects of non-IPD allowed for ages 0–4 years only): EGP19,890/QALY. *[$13,357/QALY; Base case]*<br>**2. Scenario or sensitivity analysis results:**<br>2.1. Payer (Allow inpatient pneumonia herd effects for all ages): EGP16,813/QALY. *[$11,290/QALY; -15.48%]*

	Child	72 GAVI eligible countries (49)	2005	**1. Main results:**<br>1.1. Societal with direct effects only: $112/DALY. *[$173/DALY, Base case]*<br>1.2. Societal with direct plus indirect effects: $88/DALY. *[$136/DALY; -21.39%]*
	Child	77 MICs (48)	2005	**1. Main results:**<br>1.1. Societal: $1000/DALY. *[$1541/DALY, Base]*<br>**2. Scenario or sensitivity analysis results: unclear**
	Child	Argentina (UMIC) (50)	2009	**1. Main results:**<br>1.1. Healthcare: ARS33,200/DALY. *[$210,528/DALY; Base case]*<br>1.2. Societal: ARS31,620/DALY. *[$200,509/DALY; -4.76%]*
	Child	Colombia (UMIC) (51)	2009	**1. Main results:**<br>1.1. Societal: COP3,755,251/YLG. *[$5,563/YLG; Base case]*
	Child	Thailand (UMIC) (56)	2010	**1. Main results:**<br>1.1. Societal with herd immunity: THB650,087/QALY. *[$67,218/QALY, -61.24%]*<br>**2. Scenario or sensitivity analysis results:**<br>2.1. Societal without herd immunity: THB1,677,379/QALY. *[$173,437; Base case]*
	Child	Paraguay (UMIC) (58)	2009	**1. Main results:**<br>1.1. Government: PYG19,126,694/DALY. *[$12,557/DALY; Base case]*<br>1.2. Societal: PYG9,536,030/DALY. *[$6,260/DALY, -50.15%]*
	Child	China (UMIC) (61)	2014	**1. Main results:**<br>1.1. Societal: CNY102,374/QALY. *[$34,445; Base case]*
	Child	Malaysia (UMIC) (63)	2014	**1. Main results**<br>1.1. Payer: MYR71,389/QALY. *[$61,426; Base case]*<br>1.2. Payer: MYR67,286/LYG. *[$57,896; Base case]*<br>1.3. Societal: MYR68,821/QALY. *[$59,216; -3.60%]*<br>1.4. Societal: MYR64,868/LYG. *[$55,815; -3.59%]*
	Child	Colombia (UMIC) (67)	2008	**1. Main results:**<br>1.1. Healthcare: I$1,417/DALY. [$2024/DALY; Base case]<br>1.2. Societal: I$1,301/DALY. [$1859/DALY; -8.20%]
	Child	Thailand (UMIC) (70)	2017	**1. Main results:**<br>1.1. Societal with no consideration of herd effect: THB170,437/QALY. *[$15,326/QALY; Base case]*<br>**2. Scenario or sensitivity analysis results:**<br>2.1. Societal with consideration of herd effect: Dominant
	Child	Indonesia (UMIC) (79)	2019	**1. Main results:**<br>1.1. With UNICEF price:<br>1.1.1. Healthcare: $218/QALY. *[$262/QALY; Base case]*<br>1.1.2. Payer: $178/QALY. *[$214/QALY; -18.32%]*<br>1.1.3. Societal: $210/QALY. *[$252/QALY; -3.82%]*

				1.2. With government contract price:<br>1.2.1. Healthcare: $987/QALY. *[$1,185/QALY; Base case]*<br>1.2.2. Payer: $948/QALY. *[$1,139/QALY; -3.88%]*<br>1.2.3. Societal: $979/QALY. *[$1,176/QALY; -0.76%]*
	Child	Thailand (UMIC) (81)	NR (2021)	**1. Main results:**<br>1.1. Societal with herd immunity: THB39,322/QALY. *[$3,436/QALY; Base case]*
	Child	The Netherlands (HIC) (47)	NR (2009)	**1. Main results:**<br>1.1. Societal with 10% net positive indirect effects against invasive pneumococcal disease for individuals aged 5 years or older. (with no net indirect benefits for PCV-7 and 10% net indirect benefits for PCV-10): €52,947/QALY. *[$91,036/QALY, -46.60%]*<br>**2. Scenario or sensitivity analysis results:**<br>**For indirect effects:**<br>2.1. Societal without net positive indirect effects for individuals aged 5 years or older (herd protection against vaccine serotype disease minus increases in non-vaccine serotype pneumococcal disease): €99,151/QALY. *[$170,477/QALY, Base case]*<br>2.2. Societal with 20% net positive indirect effects for individuals aged 5 years or older (herd protection against vaccine serotype disease minus increases in non-vaccine serotype pneumococcal disease): €35,146/QALY. *[$60,429/QALY; -64.55%]*<br>**For herd immunity (invasive pneumococcal disease):**<br>2.3. Societal with excluding herd effects in the analysed birth cohort for invasive pneumococcal disease. Scenarios were calculated holding all other assumptions, similar to the base case analysis (that is, with no net indirect benefits for PCV-7 and 10% net indirect benefits for PCV-10: €57,770/QALY. *[$99,328/QALY; Base case]*<br>2.4. *Societal* with 10% net positive indirect effects against invasive pneumococcal disease for individuals aged 5 years or older. (with no net indirect benefits for PCV-7 and 10% net indirect benefits for PCV-10): €52,947/QALY. *[$91,036/QALY, -8.35%]*<br>**For herd immunity (non-invasive pneumococcal disease):**<br>2.5. Societal with 10% net positive indirect effects against invasive pneumococcal disease for individuals aged 5 years or older. (with no net indirect benefits for PCV-7 and 10% net indirect benefits for PCV-10): €52,947/QALY. *[$91,036/QALY, Base case]*<br>2.6. Societal with including herd effects in the analysed birth cohort for non-invasive pneumococcal disease. Scenarios were calculated holding all other assumptions, similar to the base case analysis (that is, with no net indirect benefits for PCV-7 and 10% net indirect benefits for PCV-10): €52,211/QALY. *[$89,770/QALY; -1.39%]*<br>**For perspective:**<br>2.7. Exclusion of productivity losses (analysis from a healthcare perspective). Scenarios were calculated holding all other assumptions, similar to the base case analysis (that is, with no net indirect benefits for PCV-7 and 10% net indirect benefits for PCV-10: €53,904/QALY. [$92,681/QALY; base case]

				2.8. Societal with 10% net positive indirect effects against invasive pneumococcal disease for individuals aged 5 years or older. (with no net indirect benefits for PCV-7 and 10% net indirect benefits for PCV-10): €52,947/QALY. *[$91,036/QALY, -1.77%]*
	Child	Croatia (HIC) (60)	2014	**1. Main results:**<br>1.1. Government: Kn433,797/DALY. *[$163,681/DALY; Base case]*<br>1.2. Societal: Kn394,887/DALY. *[$148,999/DALY; -8.97%]*
	Child	Hong Kong (HIC) (63)	2014	**1. Main results**<br>1.1. Payer: HKD 362,948/QALY. *[$76,334; Base case]*<br>1.2. Payer: HKD664,400/LYG. *[$139,735; Base case]*<br>1.3. Societal: HKD317,153/QALY. *[$66,703; -12.62%]*<br>1.4. Societal: HKD580,576/LYG. *[$122,105; -12.62%]*
**PCV12 vs NV**	Child	Thailand (UMIC) (81)	NR (2021)	**1. Main results:**<br>1.1. Societal with herd immunity: Dominant
**PCV13 vs NV**	Child	Gambia (LIC) (46)	2005	**1. Main results:**<br>1.1. Societal (without indirect effects): $570/DALY. *[$2,789/DALY, Base case]*<br>**2. Scenario or sensitivity analysis results:**<br>2.1. With Serotype replacement (by 25%): $970/DALY. *[$4,746/DALY; 70.17%].*<br>2.2. With herd immunity (Assumed incidence decreased by 32%, 32%, 8%, and 18% for individuals aged 5-19 years, 20-39 years, 40-64 years, and 65 years and older): $430/DALY. *[$2,104/DALY; -24.56%].*<br>2.3. With indirect effects (Serotype replacement (increased incidence of non-primary endpoint pneumonia among vaccinated, and Herd immunity (assumed incidence decrease by 32%, 32%, 8%, and 18% for individuals aged 5-19 years, 20-39 years, 40-64 years, and 65 years and older): $550/DALY. *[$2,691/DALY; -3.51%].*
	Child	Gambia (LIC) (77)	2017	**1. Main results:**<br>1.1. Government: Dominant<br>1.2. Societal: Dominant<br>**2. Scenario or sensitivity analysis results:**<br>2.1. Government: $91/DALY. *[$408/DALY; Base case]*<br>2.2. Societal: $56/DALY. *[$251/DALY; -38.48%]*
	Child	Kenya (LMIC) (54)	2010	**1. Main results:**<br>1.1. Societal and no indirect effects: KES3,724/DALY. [$152/DALY; Base case]<br>**2. Scenario or sensitivity analysis results:**<br>2.1. Societal and indirect effects in the entire population: KES1,981/DALY. *[$81/DALY; -46.71%]*
	Child	Nigeria (LMIC) (73)	2017	**1. Main results:**<br>1.1. For the period 2014-2025 with Gavi support:<br>1.1.1. Government: N16,484/DALY. *[$227/DALY; Base case]*<br>1.1.2. Societal: N14,347/DALY. *[$198/DALY; -12.78%]*

				1.2. For the period 2026-2033 without Gavi support:<br>1.2.1. Government: N21,062/DALY. *[$291/DALY; Base case]*<br>1.2.2. Societal: N18,315/DALY. *[$253/DALY; -13.06%]*
	Child	Egypt (LMIC) (83)	2016	**1. Main results:**<br>1.1. Payer (Indirect effects of IPD allowed for all ages. Indirect effects of non-IPD allowed for ages 0–4 years only): EGP 9,284/QALY. *[$6,234/QALY; Base case]*<br>**2. Scenario or sensitivity analysis results:**<br>2.1. Payer (Allow inpatient pneumonia herd effects for all ages): EGP5,203/QALY. *[$3,494/QALY; -43.95%]*
	Child	Ghana (LMIC) (96)	2022	**1. Main results:**<br>1.1. With Gavi support period (2012-2025):<br>*1.1.1.* Government: GHS976/DALY. *[$721/DALY; Base case]*<br>1.1.2. Societal: GHS802/DALY. *[$593/DALY; -17.75%]*<br>1.2. After Gavi transition (2026–2031):<br>1.2.1. Government: GHS4,384/DALY. *[$3,239/DALY; Base case]*<br>1.2.2. Societal: GHS4,219/DALY. *[$3,117/DALY; -3.77%]*
	Child	72 GAVI eligible countries (49)	2005	**1. Main results:**<br>1.1. Societal with direct effects only: $105/DALY. *[$162/DALY, Base case]*<br>1.2. Societal with direct plus indirect effects: $77/DALY. *[$119/DALY; -26.54%]*
	Child	77 MICs (48)	2005	**1. Main results:**<br>1.1. Societal: $900/DALY. *[$1387/DALY, Base]*<br>**2. Scenario or sensitivity analysis results: unclear**
	Child	Argentina (UMIC) (50)	2009	**1. Main results:**<br>1.1. Healthcare: ARS40,508/DALY. *[$256,870/DALY, Base case]*<br>1.2. Societal: ARS38,887/DALY. *[$246,591/DALY, -4%]*
	Child	Thailand (UMIC) (56)	2010	**1. Main results:**<br>1.1. Societal with herd immunity: THB660,662/QALY. *[$68,311/QALY, -63.91%]*<br>**2. Scenario or sensitivity analysis results:**<br>2.1. Societal without herd immunity: THB1,830,716/QALY. *[$189,292; Base case]*
	Child	Paraguay (UMIC) (58)	2009	**1. Main results:**<br>1.1. Government: PYG24,341,710/DALY. *[$15,980/DALY; Base case]*<br>1.2. Societal: PYG18,163,157/DALY. *[$11,924/DALY, -25.38%]*
	Child	China (UMIC) (61)	2014	**1. Main results:**<br>1.1. Societal: CNY70,428/QALY. *[$23,696/QALY; Base case]*
	Child	China (UMIC) (62)	2015	**1. Main results:**<br>1.1. Societal: CNY 183,462/QALY. *[$61713; Base case]*
	Child	Malaysia (UMIC) (63)	2014	**1. Main results (with herd effect for PCV13 against IPD, pneumonia, and AOM)**<br>1.1. Payer: MYR21,275/QALY. *[$18,306/QALY; Base case]*

				1.2. Payer: MYR 19,810/LYG. *[$17,045/LYG; Base case]*<br>1.3. Societal: MYR16,260/QALY. *[$13,991/QALY; -23.57%]*<br>1.4. Societal: MYR15,142/LYG**.** *[$13,028/LYG; -23.57%]*<br>**2. Scenario or sensitivity analysis results**<br>**For herd effect in IPD, pneumonia, and AOM versus without:**<br>2.1. Payer with no herd effect for PCV13 against IPD, pneumonia, and AOM: MYR52,454/QALY. *[$45,134; Base case]*<br>2.2. Payer with herd effect for PCV13 against IPD, pneumonia, and AOM: MYR21,275/QALY. *[$18,306/QALY; -59.44%]*<br>2.3. Societal with no herd effect for PCV13 against IPD, pneumonia, and AOM: MYR49,887/QALY. *[$42,925/QALY; Base case]*<br>2.4. Societal with herd effect for PCV13 against IPD, pneumonia, and AOM: MYR16,260/QALY *[$13,991/QALY; -67.41%]*<br>**For herd effect in IPD, pneumonia, and AOM versus herd effect in IPD only:**<br>2.5. Payer with only herd effect for IPD: MYR47,296/QALY. *[$40,695/QALY; Base case]*<br>2.6. Payer with herd effect for PCV13 against IPD, pneumonia, and AOM: MYR21,275/QALY. *[$18,306/QALY; -55.02%]*<br>2.7. Societal with only herd effect for IPD: MYR44,962/QALY. *[$38,687/QALY; Base case]*<br>2.8. Societal with herd effect for PCV13 against IPD, pneumonia, and AOM: MYR16,260/QALY. *[$13,991/QALY; -63.84%]*
	Child	Mongolia (UMIC) (66)	2014	**1. Main results (with direct and indirect population effects)**<br>1.1. Health system (without productivity): MNT93,834/DALY. *[$224/DALY; Base case]*<br>1.2. Societal (with productivity): Dominant<br>**2. Scenario or sensitivity analysis results**<br>*2.1.* Health system (with direct population effects only): MNT 142,556/DALY. *[$341/DALY; Base case].*<br>*2.2.* Health system with direct and indirect population effects: MNT93,834/DALY. *[$224/DALY; -34.31%]*<br>2.3. Societal (with direct population effects only): MNT 34,286 /DALY. *[$82/DALY; Base case]*.<br>2.4. Societal with direct and indirect population effects: Dominant
	Child	China (UMIC) (68)	2016	**1. Main results:**<br>1.1. With herd protection:<br>1.1.1. Healthcare: CNY120,856/QALY. *[$40,300/QALY; Base case]*<br>1.1.2. Societal: CNY107,016/QALY. *[$35,685/QALY; -11.45%]*<br>1.2. Without herd protection:<br>*1.2.1.* Healthcare without herd protection: CNY137,600/QALY. *[$45,884/QALY; Base case]*<br>1.2.2. Healthcare with herd protection: CNY120,856/QALY. *[$40,300/QALY; -12.17%]*

				1.2.3. Societal without herd protection: CNY122,810/QALY. *[$40,952/QALY; Base case]*<br>1.2.4. Societal with herd protection: CNY107,016/QALY. *[$35,685/QALY; -12.86%]*
	Child	Thailand (UMIC) (70)	2017	**1. Main results:**<br>*1.1.* Societal with no consideration of herd effect: THB73,674/QALY. *[$6,624/QALY; Base case]*<br>**2. Scenario or sensitivity analysis results:**<br>2.1. Societal with consideration of herd effect: Dominant
	Child	Iran (UMIC) (71)	2014	**1. Main results:**<br>1.1. Government: IRR66,150,000/DALY. *[$19,365/DAY; Base case]*<br>1.2. Societal: IRR53,830,000/DALY. *[$15,758/DAY; -18.63%]*
	Child	Malaysia (UMIC) (74)	2018	**1. Main results:**<br>1.1. Societal: Dominant
	Child	Indonesia (UMIC) (79)	2019	**1. Main results:**<br>1.1. With UNICEF price:<br>1.1.1. Healthcare: $162/QALY. *[$195/QALY; Base case]*<br>1.1.2. Payer: $122/QALY. *[$147/QALY; -24.62%]*<br>1.1.3. Societal: $154/QALY. *[$185/QALY; -5.13%]*<br>1.2. With government contract price:<br>1.2.1. Healthcare: $747/QALY. *[$897/QALY; Base case]*<br>1.2.2. Payer: $707/QALY. *[$849/QALY; -5.35%]*<br>1.2.3. Societal: $739/QALY. *[$888/QALY; -1%]*
	Child	China (UMIC) (80)	2019	**1. Main results:**<br>1.1. Societal: CNY106,348/QALY. *[$32,300/QALY; Base]*
	Child	Thailand (UMIC) (81)	NR (2021)	**1. Main results:**<br>1.1. Societal with herd immunity: THB15,523/QALY. *[$1,357/QALY; Base case]*
	Child	The Netherlands (HIC) (47)	NR (2009)	**1. Main results:**<br>1.1. Societal with 10% net positive indirect effects against invasive pneumococcal disease for individuals aged 5 years or older. (with no net indirect benefits for PCV-7 and 10% net indirect benefits for PCV-13): € 50,042/QALY. *[$86,041/QALY, -45.43%]*<br>2. Scenario or sensitivity analysis results:<br>**For indirect effects:**<br>2.1. Societal without net positive indirect effects for individuals aged 5 years or older (herd protection against vaccine serotype disease minus increases in non-vaccine serotype pneumococcal disease): € 91,705/QALY. *[$157,674/QALY, Base case]*<br>2.2. Societal with 20% net positive indirect effects for individuals aged 5 years or older (herd protection against vaccine serotype disease minus increases in non-vaccine serotype pneumococcal disease): € 33,479/QALY. *[$57,563/QALY; -78.77%]*

				**For herd immunity (invasive pneumococcal disease):**<br>2.3. Societal with excluding herd effects in the analysed birth cohort for invasive pneumococcal disease. Scenarios were calculated holding all other assumptions, similar to the base case analysis (that is, with no net indirect benefits for PCV-7 and 10% net indirect benefits for PCV-13: € 55,055/QALY. *[$94,660/QALY; Base case]*<br>2.4. Societal with 10% net positive indirect effects against invasive pneumococcal disease for individuals aged 5 years or older. (with no net indirect benefits for PCV-7 and 10% net indirect benefits for PCV-13): € 50,042/QALY. *[$86,041/QALY, -9.11%]*<br>**For herd immunity (non-invasive pneumococcal disease):**<br>2.5. Societal with 10% net positive indirect effects against invasive pneumococcal disease for individuals aged 5 years or older. (with no net indirect benefits for PCV-7 and 10% net indirect benefits for PCV-13): € 50,042/QALY. [$86,041/QALY, Base case]<br>2.6. Societal with including herd effects in the analysed birth cohort for non-invasive pneumococcal disease. Scenarios were calculated holding all other assumptions, similar to the base case analysis (that is, with no net indirect benefits for PCV-7 and 10% net indirect benefits for PCV-13): €49,407/QALY. *[$84,949/QALY; -1.27%]*<br>**For perspective:**<br>2.7. Exclusion of productivity losses (analysis from a healthcare perspective). Scenarios were calculated holding all other assumptions, similar to the base case analysis (that is, with no net indirect benefits for PCV-7 and 10% net indirect benefits for PCV-13: € 50,938/QALY. *[$87,581/QALY; Base case]*<br>2.8. Societal with 10% net positive indirect effects against invasive pneumococcal disease for individuals aged 5 years or older. (with no net indirect benefits for PCV-7 and 10% net indirect benefits for PCV-13): € 50,042/QALY. *[$86,041/QALY, -1.76%]*
	Child	Taiwan (HIC) (53)	2009	**1. Main results:**<br>1.1. Healthcare: NT$1,218,581/LYG. *[$103,645/LYG, Base case]*<br>1.2. Societal: NT$586,117/LYG. *[$49,852; -51.90%]*
	Child	Japan (HIC) (55)	NR (2012)	**1. Main results:**<br>1.1. Societal: ¥4,495,903/QALY. *[$55,997/QALY; -50.24%]*<br>1.2. Societal: ¥14,667,948/YOLS. *[$182,690/YOLS, -50.24%]*<br>**2. Scenario or sensitivity analysis results**<br>2.1. Healthcare: ¥9,034,940/QALY. *[$112,530/QALY; Base case]*<br>2.2. Healthcare: ¥29,476,620/YOLS. *[$367,132/YOLS; Base case]*
	Child	Croatia (HIC) (60)	2014	**1. Main results:**<br>1.1. Government: Kn440,062/DALY. *[$166,045/DALY; Base case]*<br>1.2. Societal: Kn402,239/DALY. *[$151,773/DALY; -8.60%]*
	Child	Hong Kong (HIC) (63)	2014	**1. Main results (with herd effect for PCV13 against IPD, pneumonia, and AOM):**<br>1.1. Payer: Dominant<br>1.2. Payer: Dominant

				1.3. Societal: Dominant<br>1.4. Societal: Dominant<br>**2. Scenario or sensitivity analysis results:**<br>**For herd effect in IPD, pneumonia, and AOM versus without:**<br>2.1. Payer with no herd effects for PCV13 against IPD, pneumonia, and AOM: HKD 406,030 /QALY. *[$85,395/QALY; Base case]*<br>2.2. Payer with herd effect for PCV13 against IPD, pneumonia, and AOM: Dominant<br>2.3. Societal with no herd effects for PCV13 against IPD, pneumonia, and AOM: HKD 377,092 /QALY. *[$79,309/QALY; Base case]*<br>2.4. Societal with herd effect for PCV13 against IPD, pneumonia, and AOM: Dominant<br>**For herd effect in IPD, pneumonia, and AOM versus herd effect in IPD only:**<br>2.5. Payer with Only herd effect for IPD: HKD 174,406 /QALY. *[$36,681/QALY; Base case]*<br>2.6. Payer with herd effect for PCV13 against IPD, pneumonia, and AOM: Dominant<br>2.7. Societal with Only herd effect for IPD: HKD138,725 /QALY. *[$29,176/QALY; Base case]*<br>2.8. Societal with herd effect for PCV13 against IPD, pneumonia, and AOM: Dominant
	Child	Portugal (HIC) (64)	2015	**1. Main results:**<br>1.1. Societal with herd effect for the adult population: Dominant<br>**2. Scenario or sensitivity analysis results:**<br>**With herd effect versus without**<br>2.1. Societal with exclusion of herd effect: €89,539/LYG. *[$183,290/LYG; Base case]*<br>2.2. Societal with herd effect for the adult population: Dominant<br>**With herd effect in 65+ population versus herd effect in 65+ population -50%**<br>2.3. Societal with herd effect in 65+ population −50%: €1,401/LYG. *[$2,868/LYG; Base case]*<br>2.4. Societal with herd effect for the adult population: Dominant<br>**For perspective**<br>2.5. Exclusion of indirect costs (Healthcare perspective): €2,260/LYG. *[$4,626/LYG; Base case]*<br>2.6. Societal with herd effect for the adult population: Dominant
	Child	Global (69)	2015	**1. Main results:**<br>1.1. Societal: I$724/DALY. [$933/DALY]
	Adult	China (UMIC) (84)	2019	**1. Main results:**<br>1.1. Societal: CNY63,510/QALY. *[$19,289/QALY; Base case]*
	Adult	Germany (HIC) (52)	2010	**1. Main results:**<br>1.1. Societal: Dominant
	Adult	Netherlands (HIC) (59)	2012	**1. Main results:**<br>1.1. Societal: €8,647/QALY. *[$14,490/QALY; -2.67%]*<br>1.2. Societal: €7,649/LYG. *[$12,817/LYG;-2.67%]*<br>**2. Scenario or sensitivity analysis results:**<br>2.1. Healthcare: €8,884/QALY. *[$14,887/QALY, Base case]*

				2.2. Healthcare: €7,859/LYG. *[$13,169/LYG; Base case]*
	Adult	Portugal (HIC) (72)	2013	**1. Main results with herd effects from the pediatric vaccination program (assumed an immunization rate of 100% among adults + lost productivity):**<br>1.1. Societal: €17,746/QALY. [$37,321/QALY]<br>1.2. Societal: €11,082/LYG. [$23,306/LYG]<br>**2. Scenario or sensitivity analysis results:**<br>**For perspective:**<br>2.1. No indirect costs (healthcare perspective):<br>2.1.1. €18,224/QALY. *[$38,327/QALY; Base case]*<br>2.1.2. €11,380/LYG. *[$23,933/LYG; Base case]*<br>2.2. Societal with lost productivity:<br>2.2.1. €17,746/QALY. *[$37,321/QALY; -2.62%]*<br>2.2.2. €11,082/LYG. [$23,306/LYG; -2.62%]<br>2.3. Societal Inclusion of travel costs (50% incurs in travel costs for the vaccinated population):<br>2.3.1. €18,526/QALY. *[$38,962/QALY; 1.66%]*<br>2.3.2. €11,570/LYG. *[$24,333/LYG; 1.67%]*<br>**For herd effects**<br>2.4. **Societal with no herd effect from the pediatric vaccination program:**<br>2.4.1. €17,270/QALY. *[$36,320/QALY; Base case]*<br>2.4.2. €10,802/LYG. *[$22,718/LYG; Base case]*<br>2.5. **Societal with herd effects from the pediatric vaccination program (assumed an immunization rate of 100% among adults + lost productivity):**<br>2.5.1. €17,746/QALY. *[$37,321/QALY; 2.76%]*<br>2.5.2. €11,082/LYG. *[$23,306/LYG; 2.59%]*<br>2.6. **Societal with herd effects from the pediatric vaccination program (assumed an immunization rate of +50% among adults):**<br>2.6.1. €17,991/QALY. *[$37,837/QALY; 4.18%]*<br>2.6.2. €11,226/LYG. *[$23,609/LYG; 3.92%]*
	Adult	Netherlands (HIC) (75)	2017	**1. Main results:**<br>1.1. Societal with future unrelated medical costs: €28,540/QALY. *[$45,952/QALY; Base case]*.<br>1.2. Societal with future unrelated medical costs and future non-medical consumption costs: €45,691/QALY. *[$73,567/QALY; 60.10%]*.
	Adult	Japan (HIC) (76)	2021	**1. Main results:**<br>1.1. Payer:<br>1.1.1. ¥1,139,438/QALY. *[$13,486/QALY; Base case]*<br>1.1.2. ¥996,973/LYG. *[$11,800/LYG; Base case]*<br>1.2. Societal:<br>1.2.1. ¥1,356,218/QALY. *[$16,052/QALY; 19.03%]*<br>1.2.2. ¥1,186,649/LYG. *[$14,045/LYG; 19.03%]*

**PCV13 vs PCV10**				
	Child	Philippines (LMIC) (78)	2020	**1. Main results:**<br>*1.1.* Societal: Dominant<br>**2. Scenario or sensitivity analysis results:**<br>2.1. Healthcare: Dominant
	Child	Egypt (LMIC) (83)	2016	**1. Main results:**<br>1.1. Payer (Indirect effects of IPD allowed for all ages. Indirect effects of non-IPD allowed for ages 0–4 years only): EGP1,744/QALY. *[$1,171/QALY; Base case]*<br>**2. Scenario or sensitivity analysis results:**<br>2.1. Payer (Allow inpatient pneumonia herd effects for all ages): EGP712/QALY. *[$478/QALY; -59.18%]*
	Child	Argentina (UMIC) (50)	2009	**1. Main results:**<br>1.1. Healthcare: ARS104,144/DALY. *[$660,400/DALY, Base case]*<br>1.2. Societal: ARS102,172/DALY. *[$647,895/DALY, -1.89%]*
	Child	Colombia (UMIC) (51)	2009	**1. Main results:**<br>1.1. Societal: COP19,452,893/YLG. *[$28,819/YLG; Base case]*
	Child	Paraguay (UMIC) (58)	2009	**1. Main results:**<br>1.1. Government: PYG60,499,157/DALY. *[$39,717/DALY; Base case]*<br>1.2. Societal: PYG77,957,045/DALY. *[$51,178/DALY, 28.86%]*
	Child	Malaysia (UMIC) (63)	2014	**1. Main results (adjusted by serotype coverage and herd effect for PCV13 only)**<br>1.1. Payer: Dominant<br>1.2. Payer: Dominant<br>1.3. Societal: Dominant<br>1.4. Societal: Dominant
	Child	Malaysia (UMIC) (74)	2018	**1. Main results:**<br>1.1. Societal with indirect effects of IPDs: Dominant<br>**2. Scenario or sensitivity analysis results:**<br>**For perspective:**<br>2.1. Payer perspective: Dominant<br>2.2. Societal with indirect effects of IPDs: Dominant<br>**For indirect effect in IPD only versus IPD + pneumonia**<br>2.3. Societal with indirect effects of IPDs: Dominant<br>2.4. Societal with indirect effects of pneumonia included (both vaccines): Dominant
	Child	Colombia (UMIC) (87)	2022	**1. NR**
	Child	Netherlands (HIC) (57)	2012	**1. Main results:**<br>1.1. Societal with direct effects, IPD only: €670,000/QALY. *[$1,122,760/QALY; Base case]*<br>1.2. Societal with direct and indirect effects, IPD only: €14,500/QALY. *[$24,298/QALY; -97.84%]*

				1.3. Societal with direct and indirect effects, IPD + noninvasive disease: €12,700/QALY. *[$21,282; -98.10%]*<br>**2. Scenario or sensitivity analysis results (for direct + indirect effects IPD + noninvasive disease):**<br>2.1. Societal with herd protection and replacement based on UK data: €4,700/QALY. *[$7,876; -99.30%]*<br>2.2. Societal with reduced indirect effects based on UK-data (25%): €25,500/QALY. *[$42,732/QALY; -96.19%]*
	Child	Croatia (HIC) (60)	2014	**1. Main results:**<br>1.1. Government: Kn481,869/DALY. *[$181,819/DALY; Base case]*<br>1.2. Societal: Kn451,271/DALY. *[$170,274/DALY; -6.35%]*
	Child	Hong Kong (HIC) (63)	2014	**1. Main results (with herd effect for PCV13 against IPD, pneumonia, and AOM):**<br>1.1. Payer: Dominant<br>1.2. Payer: Dominant<br>1.3. Societal: Dominant<br>1.4. Societal: Dominant<br>**2. Scenario or sensitivity analysis results:**<br>**For herd effect in IPD, pneumonia, and AOM versus without:**<br>2.1. Payer with no herd effects for PCV13 against IPD, pneumonia, and AOM: HKD506,904/QALY. *[$10,6611/QALY; Base case]*<br>2.2. Payer with herd effect for PCV13 against IPD, pneumonia, and AOM: Dominant<br>2.3. Societal with no herd effects for PCV13 against IPD, pneumonia, and AOM: HKD506,951/QALY. *[$106,621/QALY; Base case]*<br>2.4. Societal with herd effect for PCV13 against IPD, pneumonia, and AOM: Dominant<br>**For herd effect in IPD, pneumonia, and AOM versus herd effect in IPD only:**<br>2.5. Payer with Only herd effect for IPD: HKD73,137/QALY. *[$15,382/QALY; Base case]*<br>2.6. Payer with herd effect for PCV13 against IPD, pneumonia, and AOM: Dominant<br>2.7. Societal with Only herd effect for IPD: HKD73,145/QALY. *[$15,384/QALY; Base case]*<br>2.8. Societal with herd effect for PCV13 against IPD, pneumonia, and AOM: Dominant
	Child	Germany (HIC) (65)	2017	**1. Main results with indirect effects (herd immunity + serotype replacement):**<br>1.1. German statutory health insurance (Excluding co-payments and productivity cost): €3,368/QALY. *[$5,461/QALY; Base case]*<br>1.2. German statutory health insurance (Excluding co-payments and productivity cost): €1,882/LYG. *[$3,051/LYG; Base case]*<br>1.3. Societal: €9,826/QALY. *[$15,930/QALY; 191.70%]*<br>1.4. Societal: €5,490/LYG. *[$8,901/LYG; 191.74%]*<br>**2. Scenario or sensitivity analysis results (No indirect effects):**<br>2.1. German statutory health insurance (No indirect effects): €284,624/LYG. *[$461,462/LYG; Base case]*

				2.2. German statutory health insurance (with indirect effects): €1,882/LYG. *[$3,051/LYG; -99.34%]*<br>2.3. Societal (No indirect effects): €485,944/LYG. *[$787,862/LYG; Base case]*<br>2.4. Societal with indirect effects: €5,490/LYG. *[$8,901/LYG; -98.87%]*
**PCV15 vs NV**	Adult	Switzerland (HIC) (82)	2020	**1. Main results:**<br>1.1. Healthcare payer: CHF17,866/QALY. *[$16,385/QALY; Base case]*<br>1.2. Societal: CHF15,616/QALY. *[$14,322/QALY; -12.60%]*<br>**2. Scenario or sensitivity analysis results:**<br>2.1. Healthcare payer: CHF5,491/QALY. *[$5,036/QALY; Base case]*<br>2.2. Societal: CHF2,035/QALY. *[$1,866/QALY; -62.95%]*<br>2.3. Analysis including AMR (18% across STs) of PCV15 versus no vaccination [b]<br>2.3.1. Healthcare payer: CHF17,800/QALY. *[$16,325/QALY; -0.37%]*<br>2.3.2. Societal: CHF15,550/QALY. *[$14,261/QALY; -0.43%]*
	Adult	Netherlands (HIC) (93)	2021	**1. Main results:**<br>*1.1.* Societal while continuing PCV10 in children: €21,429/QALY. *[$31,161/QALY; Base case]*<br>1.2. Societal while switching to PCV13 in children: €26,751/QALY. *[$38,900/QALY; 24.84%]*<br>1.3. Societal while switching to PCV15 in children: €36,000/QALY. *[$52,350/QALY; 68%]*<br>1.4. Societal while switching to PCV20 in children: €36,000/QALY. *[$52,350/QALY; 68%]*
**PCV15 vs PCV13**	Child	United States (HIC) (85)	2021	**1. Main results:**<br>1.1. Societal with herd immunity: Dominant<br>**2. Scenario or sensitivity analysis results:**<br>**For perspective:**<br>2.1. Healthcare: Dominant<br>2.2. Societal: Dominant<br>**For herd immunity:**<br>2.3. Societal with no herd immunity: Dominant<br>2.4. Societal with herd immunity: Dominant
	Child	United States (HIC) (88)	2021	**1. Main results with indirect effects (assumed decline in pneumococcal disease due to serotypes 22F and 33F by removing 7.8% of serotype 22F and 33F pneumococcal disease each year among those who are unvaccinated):**<br>1.1. Societal with indirect effects: Dominant<br>1.2. Societal with indirect effects: Dominant<br>**2. Scenario or sensitivity analysis results without indirect effects (removing indirect effects from PCV15):**<br>2.1. Societal without indirect effects: Dominant<br>2.2. Societal without indirect effects: Dominant
	Child	Japan (HIC) (89)	2022	**1. Main results:** (indirect effects on IPD only and were applied to serotype 22 F and 33 F IPD, assuming an annual incidence rate reduction of 7.8% for the first five years)

				1.1. Societal: Dominant<br>1.2. Societal: Dominant<br>**2. Scenario or sensitivity analysis results:**<br>**For perspective**<br>2.1. Healthcare: Dominant<br>*2.2.* Healthcare: Dominant<br>**For herd immunity**<br>2.3. Societal (without herd immunity): Dominant<br>2.4. Societal with indirect effect: Dominant
	Child	United Kingdom (HIC) (90)	2021	**1. Main results:**<br>1.1. UK National Health Service perspective (PCV15 2+1 vs PCV13): GBP313,229/QALY. *[$521,013/QALY; Base case]*
	Child	Japan (HIC) (100)	2022	**1. Main results with herd effect as a reduction in disease incidence over time against serotypes unique to PCV15 versus PCV13 as they provide maximum protection for the entire population:**<br>1.1. Healthcare (with herd effect): Dominant<br>1.2. Societal (with herd effect): Dominant<br>**2. Scenario or sensitivity analysis results:**<br>2.1. Healthcare (indirect effects excluded): Dominant<br>2.2. Healthcare (with herd effect): Dominant<br>2.3. Societal (indirect effects excluded): Dominant<br>2.4. Societal (with herd effect): Dominant
**PCV20 vs NV**	Adult	Netherlands (HIC) (93)	2021	**1. Main results:**<br>*1.1.* Societal while continuing PCV10 in children: €9,051/QALY. *[$13,162/QALY; Base case]*<br>1.2. Societal while switching to PCV13 in children: €10,228/QALY. *[$14,873/QALY; 13%]*<br>1.3. Societal while switching to PCV15 in children: €11,173/QALY. *[$16,247/QALY; 23.44%]*<br>1.4. Societal while switching to PCV20 in children: €22,550/QALY. *[$32,791/QALY; 149.13%]*<br>**2. Scenario or sensitivity analysis results:**<br>2.1. Societal with 40% indirect protection from PCV20 in children (instead of 80%): €13,883/QALY. *[$20,188/QALY; 53.38%]*
	Adult	United States (HIC) (92)	NR (2023)	**1. Main results:**<br>1.1. Non-Black Population<br>1.1.1. Societal:<br>1.1.1.1. aged 50/65 years: $62,083/QALY. [$63,916/QALY, Base case]<br>1.2. Black Population<br>1.2.1. Societal:<br>1.2.1.1. aged 50/65 years: Dominant
**PCV20 vs PCV13**	Child	Argentina (UMIC) (98)	2023	**1. Main results:**<br>1.1. Healthcare: Dominant

				**2. Scenario or sensitivity analysis results:**<br>Societal: Dominant
	Child	Canada (HIC) (86)	2022	**1. Main results**:<br>1.1. Payer (healthcare): Dominant<br>1.2. Societal: Dominant<br>**2. Scenario or sensitivity analysis results:**<br>2.1. Cross-protection for 6C for PCV13 and PCV20<br>2.1.1. Payer (healthcare): Dominant<br>2.2. Cross-protection for 15C for PCV20<br>2.2.1. Payer (healthcare): Dominant
	Child	United Kingdom (HIC) (90)	2021	**1. Main results:**<br>1.1. UK National Health Service perspective: Dominant<br>**2. Scenario or sensitivity analysis results:**<br>2.1. Societal: Dominant
	Child	United States (HIC) (99)	2022	1. Main results:<br>1.1. Healthcare: Dominant<br>1.2. Healthcare: Dominant<br>1.3. Societal: Dominant<br>1.4. Societal: Dominant
	Child	Japan (HIC) (100)	2022	**1. Main results with herd effect as a reduction in disease incidence over time against serotypes unique to PCV20 versus PCV13 as they provide maximum protection for the entire population:**<br>1.1. Healthcare (with herd effect): Dominant<br>1.2. Societal (with herd effect): Dominant<br>**2. Scenario or sensitivity analysis results:**<br>2.1. Healthcare (indirect effects excluded): ¥117,031/QALY. [$1,382/QALY; Base case]<br>2.2. Healthcare (with herd effect): Dominant<br>2.3. Societal (indirect effects excluded): Dominant<br>2.4. Societal (with herd effect): Dominant
	Child	Germany (HIC) (101)	2022	**1. Main results:**<br>1.1. Societal: Dominant<br>**2. Scenario or sensitivity analysis results:**<br>2.1. Payer perspective: Dominant
**PCV20 vs PCV15**	Child	Argentina (UMIC) (98)	2023	**1. Main results:**<br>1.1. Healthcare: Dominant<br>**2. Scenario or sensitivity analysis results:**<br>2.1. Societal: Dominant
	Child	Canada (HIC) (86)	2022	**1. Main results**:<br>1.1. Payer (healthcare): Dominant

				1.2. Societal: Dominant<br>**2. Scenario or sensitivity analysis results:**<br>2.1. Cross-protection for 6C for PCV15 and PCV20<br>2.1.1. Payer (healthcare): Dominant<br>2.2. Cross-protection for 15C for PCV20<br>2.2.1. Payer (healthcare): Dominant
	Child	United Kingdom (HIC) [c] (90)	2021	**1. Main results:**<br>1.1. UK National Health Service perspective: Dominant<br>**2. Scenario or sensitivity analysis results:**<br>2.1. Societal: Dominant
	Child	Sweden (HIC) (94)	2023	**1. Main results:**<br>1.1. Healthcare: Dominant<br>**2. Scenario or sensitivity analysis results:**<br>2.1. Societal (burden from pediatric population): Dominant<br>2.2. Direct effects: Cross-protection for 6C (93.7%) and 15C (88.7%): Dominant<br>*2.3.* Reduce indirect effects by half: Dominant<br>2.4. Extending time to realize indirect effects (i.e., accrual in the first two years = 0%): Dominant
	Child	Germany (HIC) (95)	2023	**1. Main results:**<br>1.1. Societal (with herd immunity and serotype replacement): €503,620/QALY. *[$672,060/QALY]*<br>**2. Scenario or sensitivity analysis results:**<br>**For perspective**<br>*2.1.* Healthcare: €551687/QALY. *[$736,203/QALY; Base case]*<br>2.2. Societal €503,620/QALY. *[$672,060/QALY; -8.71%]*<br>**For serotype replacement**<br>2.3. Societal (with herd protection effects and no serotype replacement): €295,248/QALY. *[$393,996/QALY; Base case]*<br>2.4. Societal (with herd immunity and serotype replacement): €503,620/QALY. *[$672,060/QALY; 70.58%]*
	Child	United States (HIC) (99)	2022	**1. Main results:**<br>1.1. Healthcare: Dominant<br>1.2. Healthcare: Dominant<br>1.3. Societal: Dominant<br>1.4. Societal: Dominant
	Child	Japan (HIC) (100)	2022	**1. Main results with herd effect as a reduction in disease incidence over time against serotypes unique to PCV20 versus PCV15 as they provide maximum protection for the entire population::**<br>1.1. Healthcare (with herd effect): Dominant

				1.2. Societal (with herd effect): Dominant<br>**2. Scenario or sensitivity analysis results:**<br>2.1. Healthcare (indirect effects excluded): ¥1,003,530/QALY. [$11,848/QALY; Base case]<br>2.2. Healthcare (with herd effect): Dominant<br>2.3. Societal (indirect effects excluded): Dominant<br>2.4. Societal (with herd effect): Dominant
	Child	Germany (HIC) (101)	2022	**1. Main results:**<br>1.1. Societal (with indirect effects): Dominant<br>**2. Scenario or sensitivity analysis results:**<br>2.1. Payer perspective: Dominant
**PCV21 vs NV**	Adult	United States (HIC) (91)	2019	**2. Main results:**<br>2.1. Non-Black Population<br>2.1.1. Healthcare:<br>2.1.1.1. aged 50 years: $82,143/QALY. [$98,651/QALY; Base case]<br>2.1.1.2. aged 50/65 years: $159,167/QALY. [$191,154/QALY; Base case]<br>2.1.2. Societal:<br>2.1.2.1. aged 50 years: Dominant<br>2.1.2.2. aged 50/65 years: $21,500/QALY. [$25,820/QALY; -86.49%]<br>2.2. Black Population<br>2.2.1. Healthcare:<br>2.2.1.1. aged 50 years: $47,647/QALY. [$57,222/QALY; -42%]<br>2.2.1.2. aged 50/65years: $71,739/QALY. [$86,156/QALY; -54.93%]<br>2.2.2. Societal:<br>2.2.2.1. aged 50 years: Dominant<br>2.2.2.2. aged 50/65 years: Dominant
	Adult	Netherlands (HIC) (93)	2021	1. Main results:<br>1.1. Societal while continuing PCV10 in children: €6,352/QALY. [$9,237/QALY; Base case]<br>1.2. Societal while switching to PCV13 in children: €6,750/QALY. [$9,816/QALY; 6.27%]<br>1.3. Societal while switching to PCV15 in children: €7,007/QALY. [$10,189/QALY; 10.31%]<br>1.4. Societal while switching to PCV20 in children: €7,876/QALY. [$11,453/QALY; 23.99%]
**PCV21 vs PCV20**	Adult	United States (HIC) (91)	2019	**1. Main results:**<br>1.1. Non-Black Population<br>1.1.1. Healthcare:<br>1.1.1.1. aged 50 years: Dominant<br>1.1.1.2. aged 50/65 years: $50,769/QALY. [$60,972/QALY; Base case]<br>1.1.2. Societal:<br>1.1.2.1. aged 50 years: Dominant<br>1.1.2.2. aged 50/65 years: Dominant<br>1.2. Black Population

				1.2.1. Healthcare:<br>1.2.1.1. aged 50 years: Dominant<br>1.2.1.2. aged 50/65years: $33,125/QALY. [$39,782/QALY; -34.75]<br>1.2.2. Societal:<br>1.2.2.1. aged 50 years: Dominant<br>1.2.2.2. aged 50/65 years: Dominant
	Adult	Japan (HIC) (97)	2023	**1. Main results:**<br>1.1. Payer: Dominant<br>1.2. Societal: Dominant
	Adult	United States (HIC) (102)	2023	**1. Main results:**<br>1.1. Societal without herd immunity**:** Dominant<br>**2. Scenario or sensitivity analysis results:**<br>**For perspective**<br>2.1. Healthcare perspective: $3,873/QALY. *[$3,960/QALY; Base case]*<br>2.2. Societal without herd immunity: Dominant<br>**For herd immunity**<br>2.3. Societal without herd immunity: Dominant<br>Societal with herd immunity from pediatric vaccination with PCV20: Dominant

[a] ICERs were recalculated in 2024 United States dollars (USD), adjusting the values by purchasing power parity

[b] Base-case: healthcare payer and societal in main results, respectively.

[c] Comparison: PCV20 2+1 versus PCV15 2+1.

Abbreviations: ARS, Argentine Pesos; AOM, acute otitis media; CHF, Swiss francs; CNY, Chinese Yuan Renminbi; COP, Colombian pesos; DALYs, Disability-adjusted life years; EGP, Egyptian Pound; GBP, UK pounds; GHS, Ghanaian Cedi; HIC, high-income countries; HKD, Hong Kong Dollar; I$, international dollars; IPD, invasive pneumococcal disease; IRR, Iranian rials; KES, Kenyan Shilling; Kn, Croatian Kuna; LIC, low-income countries; LMIC, lower-middle-income countries; LYG, life year gain; MNT, Mongolian tugrik; MYR, Malaysian Ringgit; N, naira; NR: Not reported; NT, Taiwan New Dollar; PCVs, pneumococcal conjugate vaccines; PYG, Paraguayan Guarani; QALY, Quality-adjusted life year; ST, serotype; THB, Thai Baht; UMIC, upper-middle-income countries; YOLS, years of life saved; ¥, Japanese yen; €, Euro; $, United States dollar;
