# Supplementary material for: Consideration of intersectoral costs and benefits in health economic evaluations of pneumococcal conjugate vaccines: a systematic review
Source: Int J Technol Assess Health Care. 2026 Jul 17;42(1):e59. doi: 10.1017/S0266462326103912 (PMC13434861; doi:10.1017/S0266462326103912)
Supplement: Supplementary file 1 [file S0266462326103912sup001.zip › Supplemental_File_2.pdf]

Supplemental file 2: Search strategies

MEDLINE (Ovid)			
No.	Search strings	Date	Results
1	(Streptococcus pneumoniae/ OR exp Pneumococcal Infections/ OR (Streptococcus pneumoniae OR "s. pneumoniae" OR Pneumococc* Infection* OR Pneumococc* disease* OR IPD OR pneumococc* pneumon* OR pneumococc* sepsis OR pneumococc* sinusit* OR pneumococc* meningit* OR pneumococc* empyema OR pneumococc* otitis media OR bacteraemic pneumon*).ti,ab,kw)	01.01.2009-31.12.2024	24,140
2	(conjugate* AND (vaccin* OR immun*).ti,ab,kw)	01.01.2009-31.12.2024	27,037
3	("Costs and Cost Analysis"/ OR exp "cost of illness"/ OR exp health expenditures/ OR exp health care costs/ OR (cost OR costs OR financ* OR expen* OR economic burden OR (burden* adj2 illness) OR economic OR cost-effectiveness OR cost-benefit OR cost-utility).ti,ab,kw)	01.01.2009-31.12.2024	966,064
	1 AND 2 AND 3	01.01.2009-31.12.2024	623
EMBASE (Ovid)			
No.	Search strings	Date	Results
1	(Streptococcus pneumoniae/ OR exp Pneumococcal Infection/ OR (Streptococcus pneumoniae OR "s. pneumoniae" OR Pneumococc* Infection* OR Pneumococc* disease* OR IPD OR pneumococc* pneumon* OR pneumococc* sepsis OR pneumococc* sinusit* OR pneumococc* meningit* OR pneumococc* empyema OR pneumococc* otitis media OR bacteraemic pneumon*).ti,ab,kw)	01.01.2009-31.12.2024	48,619
2	(conjugate* AND (vaccin* OR immun*).ti,ab,kw)	01.01.2009-31.12.2024	43,448
3	("Costs and Cost Analysis"/ OR "cost of illness"/ OR health expenditures/ OR health care costs/ OR (cost OR costs OR financ* OR expen* OR "economic burden" OR (burden* adj2 illness) OR "economic" OR cost-effectiveness OR cost-benefit OR cost-utility).ti,ab,kw)	01.01.2009-31.12.2024	1,335,555
	1 AND 2 AND 3	01.01.2009-31.12.2024	899
EconLit (Ebesco)			
No.	Search strings	Date	Results
1	(MH "Streptococcus pneumoniae" OR "Streptococcus pneumoniae" OR "s. pneumoniae" OR Pneumococc* Infection* OR Pneumococc*	01.01.2009-31.12.2024	62

	disease* OR IPD OR pneumococc* pneumon* OR pneumococc* sepsis OR pneumococc* sinusit* OR pneumococc* meningit* OR pneumococc* empyema OR pneumococc* otitis media OR bacteraemic pneumon*)		
2	(conjugate* AND (vaccin* OR immun*))	01.01.2009-31.12.2024	13
3	(MH "Costs and Cost Analysis" OR MH "Cost of Illness" OR MH "Health Expenditures" OR MH "Health Care Costs" OR cost OR costs OR financ* OR expen* OR "economic burden" OR (burden* NEAR/2 illness) OR "economic" OR cost-effectiveness OR cost-benefit OR cost-utility)	01.01.2009-31.12.2024	636,401
	1 AND 2 AND 3	01.01.2009-31.12.2024	5
Web of Science			
No.	Search strings	Date	Results
1	(TS=(Streptococcus pneumoniae OR "s. pneumoniae" OR Pneumococc* Infection* OR Pneumococc* disease* OR IPD OR pneumococc* pneumon* OR pneumococc* sepsis OR pneumococc* sinusit* OR pneumococc* meningit* OR pneumococc* empyema OR pneumococc* otitis media OR bacteraemic pneumon*))	01.01.2009-31.12.2024	34,352
2	(TS=(conjugate* AND (vaccin* OR immun*)))	01.01.2009-31.12.2024	34,866
3	(TS=("Costs and Cost Analysis" OR "cost of illness" OR health expenditures OR health care costs OR cost OR costs OR financ* OR expen* OR "economic burden" OR (burden* NEAR/2 illness) OR economic OR cost-effectiveness OR cost-benefit OR cost-utility))	01.01.2009-31.12.2024	3,210,620
	1 AND 2 AND 3	01.01.2009-31.12.2024	960
CINAHL (Ebesco)			
No.	Search strings	Date	Results
1	(MH "Streptococcus pneumoniae" OR "s. pneumoniae" OR Pneumococc* Infection* OR Pneumococc* disease* OR IPD OR pneumococc* pneumon* OR pneumococc* sepsis OR pneumococc* sinusit* OR pneumococc* meningit* OR pneumococc* empyema OR pneumococc* otitis media OR bacteraemic pneumon*)	01.01.2009-31.12.2024	4,006
2	(conjugate* AND (vaccin* OR immun*))	01.01.2009-31.12.2024	3,136
3	(MH "Costs and Cost Analysis" OR MH "Cost of Illness" OR MH "Health Expenditures" OR MH	01.01.2009-31.12.2024	340,630

	"Health Care Costs" OR cost OR costs OR financ* OR expen* OR "economic burden" OR (burden* N2 illness) OR "economic" OR cost-effectiveness OR cost-benefit OR cost-utility)		
	1 AND 2 AND 3	01.01.2009-31.12.2024	129
PsycINFO (Ebesco)			
No.	Search strings	Date	Results
1	(MH "Streptococcus pneumoniae" OR "s. pneumoniae" OR Pneumococc* Infection* OR Pneumococc* disease* OR IPD OR pneumococc* pneumon* OR pneumococc* sepsis OR pneumococc* sinusit* OR pneumococc* meningit* OR pneumococc* empyema OR pneumococc* otitis media OR bacteraemic pneumon*)	01.01.2009-31.12.2024	726
2	(conjugate* AND (vaccin* OR immun*))	01.01.2009-31.12.2024	344
3	(MH "Costs and Cost Analysis" OR MH "Cost of Illness" OR MH "Health Expenditures" OR MH "Health Care Costs" OR cost OR costs OR financ* OR expen* OR "economic burden" OR (burden* N2 illness) OR "economic" OR cost-effectiveness OR cost-benefit OR cost-utility)	01.01.2009-31.12.2024	240,696
	1 AND 2 AND 3	01.01.2009-31.12.2024	9
NHS EED			
No.	Search strings	Date	Results
1	MeSH DESCRIPTOR Streptococcus pneumoniae EXPLODE ALL TREES	2009-2015	29
2	MeSH DESCRIPTOR Pneumococcal Infections EXPLODE ALL TREES	2009-2015	169
3	(Streptococcus pneumoniae OR "s. pneumoniae" OR Pneumococc* Infection* OR Pneumococc* disease* OR IPD OR pneumococc* pneumon* OR pneumococc* sepsis OR pneumococc* sinusit* OR pneumococc* meningit* OR pneumococc* empyema OR pneumococc* otitis media OR bacteraemic pneumon*):TI IN NHSEED FROM 2009 TO 2016	2009-2015	7
4	#1 OR #2 OR #3	2009-2015	174
5	(conjugate*):TI IN NHSEED FROM 2009 TO 2016	2009-2015	60
6	(vaccin* OR immun*):TI IN NHSEED FROM 2009 TO 2016	2009-2015	495
7	#5 AND #6	2009-2015	60
8	#4 AND #7	2009-2015	49
