# Supplementary material for: Consideration of intersectoral costs and benefits in health economic evaluations of pneumococcal conjugate vaccines: a systematic review
Source: Int J Technol Assess Health Care. 2026 Jul 17;42(1):e59. doi: 10.1017/S0266462326103912 (PMC13434861; doi:10.1017/S0266462326103912)
Supplement: Supplementary file 1 [file S0266462326103912sup001.zip › Supplemental_File_3.pdf]

Supplemental file 3: Studies excluded at full-text

Author	Journal	Title	DOI	Reason
Touray et al., 2011	Journal of Pharmaceutical Health Services Research	The cost effectiveness of pneumococcal conjugate vaccine in the routine infant immunisation programme of The Gambia	https://dx.doi.org/10.1111/j.1759-8893.2011.00057.x	Not adopted or included a societal perspective
Tyo et al., 2011	Vaccine	Cost-effectiveness of conjugate pneumococcal vaccination in Singapore: comparing estimates for 7-valent, 10-valent, and 13-valent vaccines	https://dx.doi.org/10.1016/j. vaccine.2011.06.091	Not adopted or included a societal perspective
Earnshaw et al., 2012	BMC Infectious Diseases	Cost-effectiveness of 2 + 1 dosing of 13-valent and 10-valent pneumococcal conjugate vaccines in Canada	https://dx.doi.org/10.1186/1471-2334-12-101	Not adopted or included a societal perspective
Smith et al., 2012	JAMA	Cost-effectiveness of adult vaccination strategies using pneumococcal conjugate vaccine compared with pneumococcal polysaccharide vaccine	https://dx.doi.org/10.1001/jama.2012.169	Not adopted or included a societal perspective
Strutton et al., 2012	The Journal of infection	Cost-effectiveness of 13-valent pneumococcal conjugate vaccine: Germany, Greece, and The Netherlands	https://dx.doi.org/10.1016 /j.jinf.2011.10.015	Not adopted or included a societal perspective
Boccalini et al., 2013	Human Vaccines & Immunotherapeutics	Cost-effectiveness of new adult pneumococcal vaccination strategies in Italy	https://dx.doi.org/ 10.4161/hv.23268	Not adopted or included a societal perspective
Gomez et al., 2013	BMC Public Health	Cost-effectiveness and cost utility analysis of three pneumococcal conjugate vaccines in children of Peru	https://dx.doi.org/10.1186/1471-2458-13-1025	Not adopted or included a societal perspective
Klok et al., 2013	Clinical Therapeutics	Cost-effectiveness of a 10- versus 13-valent pneumococcal conjugate vaccine in Denmark and Sweden	https://dx.doi.org/10.1016/j.clinthera.2012.12.006	Not adopted or included a societal perspective
Smith et al., 2013	Vaccine	Cost-effectiveness of pneumococcal conjugate vaccination in immunocompromised adults	https://dx.doi.org/10.1016/j.vaccine.2013.06.037	Not adopted or included a societal perspective
Turel et al., 2013	Value in health	Potential cost-effectiveness of pneumococcal conjugate vaccine (PCV) in Turkey	https://dx.doi.org/ 10.1016/j.jval.2013.03.1632	Not adopted or included a societal perspective
Chen et al., 2014	Advances in Therapy	Cost-effectiveness of pneumococcal vaccines for adults in the United States	https://dx.doi.org/10.1007/s12325-014-0115-y	Not adopted or included a societal perspective

Liguori et al., 2014	Human Vaccines & Immunotherapeutics	Adult immunization with 13-valent pneumococcal vaccine in Campania region, South Italy: an economic evaluation	https://dx.doi.org/10.4161/ hv.26888	Not adopted or included a societal perspective
Mezones-Holguin et al., 2014	Journal of infection in developing countries	Cost-effectiveness analysis of pneumococcal conjugate vaccines in preventing pneumonia in Peruvian children	https://dx.doi.org/10.3855/jidc.5855	Not adopted or included a societal perspective
Ordonez & Orozco, 2014	BMC Infectious Diseases	Cost-effectiveness analysis of pneumococcal conjugate vaccine 13-valent in older adults in Colombia	https://dx.doi.org/10.1186/1471-2334-14-172	Not adopted or included a societal perspective
Haasis et al., 2015	PloS One	Do Pneumococcal Conjugate Vaccines Represent Good Value for Money in a Lower-Middle Income Country? A Cost-Utility Analysis in the Philippines.	https://dx.doi.org/10.1371/journal.pone.0131156	Not adopted or included a societal perspective
Komakhidze et al., 2015	Vaccine	Cost-effectiveness of pneumococcal conjugate vaccination in Georgia.	https://dx.doi.org/10.1016/j.vaccine.2014.12.070	Not adopted or included a societal perspective
Mezones-Holguin et al., 2015	Vaccine	Cost-effectiveness analysis of 10- and 13-valent pneumococcal conjugate vaccines in Peru.	https://dx.doi.org/10.1016/j.vaccine.2014.12.039	Not adopted or included a societal perspective
Ordonez & Orozco, 2015	Cost effectiveness and resource allocation	Cost-effectiveness analysis of the available pneumococcal conjugated vaccines for children under five years in Colombia.	https://dx.doi.org/10.1186/s12962-015-0032-1	Not adopted or included a societal perspective
Sibak et al., 2015	Vaccine	Cost-effectiveness analysis of the introduction of the pneumococcal conjugate vaccine (PCV-13) in the Egyptian national immunization program, 2013.	https://dx.doi.org/10.1016/j.vaccine.2014.12.044	Not adopted or included a societal perspective
Blommaert et al., 2016	Vaccine	The cost-effectiveness of pneumococcal vaccination in healthy adults over 50: An exploration of influential factors for Belgium.	https://dx.doi.org/10.1016/j.vaccine.2016.03.003	Not adopted or included a societal perspective
van Hoek & Miller, 2016	PloS One	Cost-Effectiveness of Vaccinating Immunocompetent >=65 Year Olds with the 13-Valent Pneumococcal Conjugate Vaccine in England.	https://dx.doi.org/10.1371/journal.pone.0149540	Not adopted or included a societal perspective
Boccalini et al., 2017	Human Vaccines & Immunotherapeutics	Economic studies applied to vaccines against invasive diseases: An updated budget impact analysis of age-based pneumococcal vaccination strategies in the elderly in Italy.	https://dx.doi.org/10.1080/21645515.2017.1264827	Not adopted or included a societal perspective
Castiglia et al., 2017	Human Vaccines & Immunotherapeutics	Overall effectiveness of pneumococcal conjugate vaccines: An economic analysis of PHiD-CV and PCV-13 in the immunization of infants in Italy	https://dx.doi.org/10.1080/21645515.2017.1343773	Not adopted or included a societal perspective

Dirmesropian et al., 2017	Vaccine	Cost-effectiveness of 13-valent pneumococcal conjugate vaccine (PCV13) in older Australians.	https://dx.doi.org/10.1016/j.vaccine.2017.06.085	Not adopted or included a societal perspective
Gargano et al., 2017	Vaccine	Pneumonia prevention: Cost-effectiveness analyses of two vaccines among refugee children aged under two years, Haemophilus influenzae type b-containing and pneumococcal conjugate vaccines, during a humanitarian emergency, Yida camp, South Sudan.	https://dx.doi.org/10.1016/j.vaccine.2016.11.070	Not adopted or included a societal perspective
Castaneda-Orjuela & De la Hoz-Restrepo, 2018	Vaccine	How cost effective is switching universal vaccination from PCV10 to PCV13? A case study from a developing country.	https://dx.doi.org/10.1016/j.vaccine.2018.07.078	Not adopted or included a societal perspective
Chen et al., 2018	Vaccine	Evolution over time in the cost-effectiveness of pneumococcal conjugate vaccine (PCV13) in older Australians due to herd protection from infant vaccination.	https://dx.doi.org/10.1016/j.vaccine.2018.03.006	Not adopted or included a societal perspective
Chen et al., 2018	Vaccine	The role of timeliness in the cost-effectiveness of older adult vaccination: A case study of pneumococcal conjugate vaccine in Australia.	https://dx.doi.org/10.1016/j.vaccine.2018.01.052	Not adopted or included a societal perspective
Dorji et al., 2018	Vaccine	Towards the introduction of pneumococcal conjugate vaccines in Bhutan: A cost-utility analysis to determine the optimal policy option.	https://dx.doi.org/10.1016/j.vaccine.2018.02.048	Not adopted or included a societal perspective
Kupek & Viertel, 2018	Value in health regional issues	Postintroduction Study of Cost-Effectiveness of Pneumococcal Vaccine PCV10 from Public Sector Payer's Perspective in the State of Santa Catarina, Brazil.	https://dx.doi.org/10.1016/j.vhri.2017.12.008	Not adopted or included a societal perspective
Marbaix et al., 2018	PloS One	Cost-effectiveness of PCV13 vaccination in Belgian adults aged 65-84 years at elevated risk of pneumococcal infection.	https://dx.doi.org/10.1371/journal.pone.0199427	Not adopted or included a societal perspective
Shen et al., 2018	PloS One	Estimating the cost-effectiveness of an infant 13-valent pneumococcal conjugate vaccine national immunization program in China.	https://dx.doi.org/10.1371/journal.pone.0201245	Not adopted or included a societal perspective
Ishigami et al., 2019	American Journal of Kidney Diseases	Cost-effectiveness of Pneumococcal Vaccination Among Patients with CKD in the United States.	https://doi.org/10.1053/j.ajkd.2019.01.025	Not adopted or included a societal perspective
Krishnamoorthy et al., 2019	Vaccine	Impact and cost effectiveness of pneumococcal conjugate vaccine in India.	https://dx.doi.org/10.1016/j.vaccine.2018.12.004	Not adopted or included a societal perspective

Pugh et al., 2019	Infectious diseases and therapy	Cost-Effectiveness of the Pneumococcal Conjugate Vaccine (10- or 13-Valent) Versus No Vaccination for a National Immunization Program in Tunisia or Algeria.	https://dx.doi.org/10.1007/s40121-018-0226-x	Not adopted or included a societal perspective
Wateska et al., 2019	Human Vaccines & Immunotherapeutics	An intervention to improve pneumococcal vaccination uptake in high risk 50-64 year olds vs. expanded age-based recommendations: an exploratory cost-effectiveness analysis.	https://dx.doi.org/10.1080/21645515.2018.1564439	Not adopted or included a societal perspective
Wateska et al., 2019	Vaccine	Cost-effectiveness of adult pneumococcal vaccination policies in underserved minorities aged 50-64 years compared to the US general population.	https://doi.org/10.1016/j.vaccine.2019.01.002	Not adopted or included a societal perspective
Pugh et al., 2020	Infectious diseases and therapy	Estimating the Impact of Switching from a Lower to Higher Valent Pneumococcal Conjugate Vaccine in Colombia, Finland, and The Netherlands: A Cost-Effectiveness Analysis.	https://dx.doi.org/10.1007/s40121-020-00287-5	Not adopted or included a societal perspective
Wateska et al., 2020	Journal of the American Geriatrics Society	Cost-Effectiveness of Pneumococcal Vaccination Policies and Uptake Programs in US Older Populations.	https://dx.doi.org/10.1111/jgs.16373	Not adopted or included a societal perspective
Wateska et al., 2020	American Journal of Preventive Medicine	Pneumococcal Vaccination in Adults Aged >=65 Years: Cost-Effectiveness and Health Impact in U.S. Populations.	https://dx.doi.org/10.1016/j.amepre.2019.10.022	Not adopted or included a societal perspective
Wolff et al., 2020	Vaccine	Cost-effectiveness of pneumococcal vaccination for elderly in Sweden.	https://dx.doi.org/10.1016/j.vaccine.2020.05.072	Not adopted or included a societal perspective
Ghia et al., 2021	Infectious diseases and therapy	Estimating the Public Health and Economic Impact of Introducing the 13-Valent Pneumococcal Conjugate Vaccine or 10-Valent Pneumococcal Conjugate Vaccines into State Immunization Programs in India.	https://dx.doi.org/10.1007/s40121-021-00498-4	Not adopted or included a societal perspective
Kim et al., 2021	Human Vaccines & Immunotherapeutics	Cost-effectiveness of a national immunization program with the 13-valent pneumococcal conjugate vaccine compared with the 10-valent pneumococcal conjugate vaccine in South Korea.	https://dx.doi.org/10.1080/21645515.2020.1796426	Not adopted or included a societal perspective
Li et al., 2021	Vaccines	The Cost-Effectiveness of 13-Valent Pneumococcal Conjugate Vaccine in Seven Chinese Cities.	https://dx.doi.org/10.3390/vaccines9111368	Not adopted or included a societal perspective

Perdrizet et al., 2021	Infectious diseases and therapy	Retrospective Impact Analysis and Cost-Effectiveness of the Pneumococcal Conjugate Vaccine Infant Program in Australia.	https://dx.doi.org/10.1007/s40121-021-00409-7	Not adopted or included a societal perspective
Perdrizet et al., 2021	Human Vaccines & Immunotherapeutics	Cost-effectiveness analysis of replacing the 10-valent pneumococcal conjugate vaccine (PCV10) with the 13-valent pneumococcal conjugate vaccine (PCV13) in Brazil infants.	https://dx.doi.org/10.1080/21645515.2020.1809266	Not adopted or included a societal perspective
Smith et al., 2021	American Journal of Preventive Medicine	Higher-Valency Pneumococcal Conjugate Vaccines: An Exploratory Cost-Effectiveness Analysis in U.S. Seniors.	https://dx.doi.org/10.1016/j.amepre.2021.01.023	Not adopted or included a societal perspective
Bolanos-Diaz et al., 2022	Journal of Pharmaceutical Health Services Research	Cost-effectiveness of the 13-valent pneumococcal conjugate vaccine compared to the 10-valent vaccine in children: predictive analysis in the Ecuadorian context.	https://dx.doi.org/10.1093/jphsr/rmac040	Not adopted or included a societal perspective
Lagoubi et al., 2022	Human Vaccines & Immunotherapeutics	A cost-effectiveness analysis of PHiD-CV compared to PCV13 in a national immunization program setting in Tunisia.	https://dx.doi.org/10.1080/21645515.2022.2079305	Not adopted or included a societal perspective
Polistena et al., 2022	Vaccines	Cost-Effectiveness of Vaccination with the 20-Valent Pneumococcal Conjugate Vaccine in the Italian Adult Population.	https://dx.doi.org/10.3390/vaccines10122032	Not adopted or included a societal perspective
Smith et al., 2022	Infectious diseases and therapy	Cost-Effectiveness of Newly Recommended Pneumococcal Vaccination Strategies in Older Underserved Minority Adults in the USA.	https://dx.doi.org/10.1007/s40121-022-00669-x	Not adopted or included a societal perspective
Wateska et al., 2022	Vaccine	Cost-effectiveness of revised US pneumococcal vaccination recommendations in underserved minority adults < 65-years-old.	https://doi.org/10.1016/j.vaccine.2022.10.066	Not adopted or included a societal perspective
Gourzoulidis et al., 2023	Frontiers in public health	Health and economic outcomes of 20-valent pneumococcal conjugate vaccine compared to 15-valent pneumococcal conjugate vaccine strategies for adults in Greece.	https://dx.doi.org/10.3389/fpubh.2023.1229524	Not adopted or included a societal perspective
Huang et al., 2023	Infectious diseases and therapy	Cost-Effectiveness Analysis of the South African Infant National Immunization Program for the Prevention of Pneumococcal Disease.	https://doi.org/10.1007/s40121-023-00767-4	Not adopted or included a societal perspective
Marbaix et al., 2023	Expert review of vaccines	Cost-utility of 20-valent pneumococcal conjugate vaccine compared to no vaccination and recommended alternative vaccines among Belgian adults.	https://dx.doi.org/10.1080/14760584.2023.2273892	Not adopted or included a societal perspective

Patenaude et al., 2023	Journal of benefit-cost analysis	Sustainable Development Goal Halftime Project: Benefit-Cost Analysis Using Methods from the Decade of Vaccine Economics Model.	https://doi.org/10.1017/bca.2023.11	Not adopted or included a societal perspective
Warren et al., 2023	Vaccines	Estimating the Clinical and Economic Impact of Switching from the 13-Valent Pneumococcal Conjugate Vaccine (PCV13) to Higher-Valent Options in Greek Infants.	https://dx.doi.org/10.3390/vaccines11081369	Not adopted or included a societal perspective
Wateska et al., 2023	Vaccine	Cost-effectiveness of an in-development adult-formulated pneumococcal vaccine in older US adults.	https://dx.doi.org/10.1016/j.vaccine.2023.06.007	Not adopted or included a societal perspective
Chen et al., 2024	The Lancet. Global health	Re-evaluating the impact and cost-effectiveness of pneumococcal conjugate vaccine introduction in 112 low-income and middle-income countries in children younger than 5 years: a modelling study.	https://dx.doi.org/10.1016/S2214-109X(24)00232-8	Not adopted or included a societal perspective
Danelian et al., 2024	Vaccine	The impact and cost-effectiveness of pneumococcal immunisation strategies for the elderly in England.	https://dx.doi.org/10.1016/j.vaccine.2024.05.001	Not adopted or included a societal perspective
Ilic et al., 2024	Vaccines	The Health and Economic Effects of PCV15 and PCV20 During the First Year of Life in the US.	https://dx.doi.org/10.3390/vaccines12111279	Not adopted or included a societal perspective
Kang et al., 2024	Vaccine	Cost-effectiveness analysis of the 20-valent pneumococcal conjugate vaccine for the pediatric population in South Korea.	https://dx.doi.org/10.1016/j.vaccine.2024.05.048	Not adopted or included a societal perspective
Owusu-Edusei et al., 2024	Vaccine	Evaluating the health and economic outcomes of a PCV15 vaccination program for adults aged 65 years-and-above in Switzerland.	https://dx.doi.org/10.1016/j.vaccine.2024.04.016	Not adopted or included a societal perspective
Smith et al., 2024	American Journal of Preventive Medicine	Cost-effectiveness and public health impact of 24-valent pneumococcal conjugate vaccine compared to recommended pneumococcal vaccines in older adults.	https://dx.doi.org/10.1016/j.amepre.2024.11.014	Not adopted or included a societal perspective
Wateska et al., 2024	Journal of the American Geriatrics Society	Changes in the cost-effectiveness of pneumococcal vaccination and of programs to increase its uptake in U.S. older adults.	https://dx.doi.org/10.1111/jgs.19031	Not adopted or included a societal perspective
Rozenbaum et al., 2010	Clinical Therapeutics	Results of a cohort model analysis of the cost-effectiveness of routine immunization with 13-valent pneumococcal conjugate vaccine of those aged > or =65 years in the Netherlands.	https://dx.doi.org/10.1016/j.clinthera.2010.06.016	No relevant comparator

Rozenbaum et al., 2012	BMJ (Clinical research ed.)	Vaccination of risk groups in England using the 13 valent pneumococcal conjugate vaccine: economic analysis.	https://dx.doi.org/10.1136/bmj.e6879	No relevant comparator
Sartori et al., 2012	Journal of Epidemiology and Community Health	Cost-effectiveness of introducing the 10-valent pneumococcal conjugate vaccine into the universal immunisation of infants in Brazil.	https://dx.doi.org/10.1136/jech.2010.111880	Not relevant comparator
Carrico et al., 2021	Vaccine	Cost-benefit analysis of vaccination against four preventable diseases in older adults: Impact of an aging population.	https://doi.org/10.1016/j.vaccine.2021.07.029	Not relevant comparator
