# Supplementary material for: Consideration of intersectoral costs and benefits in health economic evaluations of pneumococcal conjugate vaccines: a systematic review
Source: Int J Technol Assess Health Care. 2026 Jul 17;42(1):e59. doi: 10.1017/S0266462326103912 (PMC13434861; doi:10.1017/S0266462326103912)
Supplement: Supplementary file 1 [file S0266462326103912sup001.zip › Supplemental_File_4.pdf]

Supplemental file 4: Additional Study characteristics

Study (year)	Perspective	Sensitivity analyses	Willingness to pay Thresholds	Funder
Kim (2010) (46)	Societal	DSA, PSA, Scenario	1-3 x GDP (1 GDP: $360)	BMGF
Rozenbaum (2010) (47)	Societal	Univariate, PSA	€50,000	Wyeth
Nakamura (2011) (48)	Societal	DSA	1-3 x GDP	Gavi
Tasslimi (2011) (49)	Societal	One-way	1-3 x GDP	Gavi
Uruena et al (2011) (50)	Health system, Societal	Scenario	1-3 x GDP ($7366-$22,000)	Wyeth
Castaneda-Orjuela (2012) (51)	Societal	DSA, PSA	1 x GDP (1 GDP: $5473.8)	MoH & EPI
Kuhlmann (2012) (52)	SHI, Societal	DSA, PSA	€20,000- €50,000	Pfizer
Wu et al (2012) (53)	Societal, Payer	DSA, PSA	1-3 x GDP<br>(3 GDP: $14,453)	National Science Council
Ayieko et al (2013) (54)	Societal	PSA	1 x GDP ($775)	Gavi Alliance
Hoshi et al (2013) (55)	Societal	One-way, PSA	¥5,000,000	None
Kulpeng et al (2013) (56)	Societal	DSA, PSA	1 x GDP (THB 100,000)	Thai Health Promotion Foundation
Vemer & Postma (2014) (57)	Societal	PSA	€50,000	Glaxo Smith Kline Co
Kieninger et al (2015) (58)	Governmental, Societal	DSA, Scenario	1-3 x GDP (1 GDP: $2,516)	None
Mangen et al (2015) (59)	Societal	Univariate, Multiway, Scenario	1-3 x GDP (1 GDP: €35,300)	Pfizer
Vucina et al (2015) (60)	Governmental, Societal	Univariate, Multivariate	1-3 x GDP ($13,530 - $40,590)	None
Maurer et al (2016) (61)	Societal	Univariate two-way, PSA	1-3 x GDP (1 GDP: $7400)	None
Mo et al (2016) (62)	Societal	Univariate, PSA	1-3 x GDP (1 GDP: $7485)	NR
Wu et al (2016) (63)	Payer, Societal	One-way, PSA, Scenario	1-3 x GDP<br>(3 GDP: $9,843, $110,099)	Pfizer
Gouveia et al (2017) (64)	Societal	Univariate	€20,000	Pfizer
Kuhlmann & von der Schulenburg (2017) (65)	SHI, Societal	Univariate, PSA	€20,000 – €30,000	NR
Sundaram et al (2017) (66)	Health system, Societal	Scenario	GDP per capita ($4,056)	WHO, Gavi
Lara et al (2018) (67)	Payer, Societal	DSA, PSA	GDP per capita PPP ($8,871)	NR
Zhou et al (2018) (68)	Healthcare, Societal	One-way, PSA	1-3 x GDP (1 GDP: $8,382)	Shanghai HFPC
Chen et al (2019) (69)	Health system, Societal	One-way, PSA	GDP	WHO, Gavi, BMGF
Dilokthornsakul et al (2019) (70)	Societal	PSA, Scenario	THB 160,000	Pfizer
Ezoji et al (2019) (71)	Societal, Government	DSA	1-3 x GDP (1 GDP: 4,526)	BMGF
Gouveia et al (2019) (72)	Societal	Univariate, PSA	€20,000	Pfizer

Idris et al (2020) (73)	Governmental, Societal	One- & two-way, PSA, Scenario	1-3 x GDP (1 GDP: $1,968)	None
Shafie et al (2020) (74)	Societal	DSA, PSA	NR	Pfizer
de Vries et al (2021) (75)	Societal	NR	€20,000	European Union commission
Igarashi et al (2021) (76)	Payer, Societal	DSA, PSA	¥ 5–6 million	Pfizer
Pecenka et al (2021) (77)	Government, Societal	PSA, Scenario	NR	Gavi, BMGF, Pfizer, MRC
Perdrizet et al (2021) (78)	Societal, Payer	Scenario	PHP 150,000	Pfizer
Suwantika et al (2021) (79)	Societal, Payer	DSA	1-3 x GDP (1 GDP: $3,859)	Clinton Health Access Initiative
Wang et al (2021) (80)	Societal	One-way, PSA	3 x GDP ($30,828)	NSFC
Chaiyakunapruk (2022) (81)	Societal	One-way, PSA	THB 160,000	BMGF
Deb et al (2022) (82)	Payer, Societal	DSA, PSA, Scenario	1 x GDP. (1 GDP: CHF 84,769)	Merck, Sharp & Dohme
Sevilla et al (2022) (83)	Payer, Societal	DSA, Multivariate PSA	1-3 x GDP (1 GDP: $3,479)	Pfizer
Guo et al (2023) (84)	Societal	One-way, PSA	1 x GDP ($10,264)	BMGF
Huang et al (2023) (85)	Societal, Healthcare	One-way, Scenario	NR	Merck, Sharp & Dohme
Lytle et al (2023) (86)	Payer, Societal	DSA, PSA, Scenario	NR	Pfizer
Ordonez (2023) (87)	Societal	DSA, PSA	NR	Pfizer
Prasad et al (2023) (88)	Societal	Univariate, Multivariate	NR	None
Tajima et al (2023) (89)	Societal, Payer	DSA, PSA, Scenario	NR	Merck, Sharp & Dohme
Wilson et al (2023) (90)	Payer, Societal	One-way, Scenario	GBP 20,000	Pfizer
Altawalbeh et al (2024) (91)	Healthcare, Societal	One-way, PSA	$100,000	NIAID
Altawalbeh et al (2024) (92)	Societal	One-way, PSA	$100,000	NIAID
de Boer et al (2024) (93)	Societal	One-way, PSA	€20,000	Netherlands MoH
Fridh et al (2024) (94)	Payer, Societal	DSA, PSA, Scenario	SEK 300,000	Pfizer
Huang et al (2024) (95)	Societal	DSA, PSA, Scenario	€180,000	Merck, Sharp & Dohme
Ibrahim et al (2024) (96)	Governmental, Societal	Two-way, PSA, Scenario	0.5 × GDP. (1 GDP: $2,445)	None
Mueller et al (2024) (97)	Payer, Societal	DSA, PSA	¥5 million	Merck, Sharp & Dohme
Rey-Ares et al (2024) (98)	Health system, Societal	DSA, PSA, Scenario	1 GDP $13,686	Pfizer
Rozenbaum et al (2024) (99)	Healthcare, Societal	DSA, PSA, Scenario	NR	Pfizer
Shinjoh et al (2024) (100)	Payer, Societal	One-way, PSA, Scenario	¥5 million	Pfizer
Ta et al (2024) (101)	Societal	DSA, PSA, Scenario	NR	Pfizer
Yi et al (2024) (102)	Healthcare, Societal	DSA, PSA, Scenario	NR	Merck, Sharp & Dohme

Abbreviations: BMGF, Bill and Melinda Gates Foundation; CHF, Swiss francs; DSA, deterministic sensitivity analysis; EPI, expanded programme on immunization; GAVI, global alliance for vaccine and immunization; GBP, UK pounds; GDP, gross domestic product; HFPC, Health and Family Planning Commission; MoH, ministry of health; MRC, Medical Research Council; NIAID, National Institute of Allergy and infectious disease; NR, not reported; NSFC, National Natural Science Foundation of China; PHP, Philippine Pesos; PPP, purchasing power parity; PSA, probabilistic sensitivity analysis; SEK, Swedish Krona; THB, Thai Baht; WHO, world health organization; €, Euro; $, United States dollar; ¥, Japanese Yen
